# Overexpression of an Arabidopsis cysteine-rich receptor-like protein kinase, CRK5, enhances abscisic acid sensitivity and confers drought tolerance

**DOI:** 10.1093/jxb/erw266

**Published:** 2016-07-12

**Authors:** Kai Lu, Shan Liang, Zhen Wu, Chao Bi, Yong-Tao Yu, Xiao-Fang Wang, Da-Peng Zhang

**Affiliations:** Center for Plant Biology, School of Life Sciences, Tsinghua University, Beijing 100084, China

**Keywords:** ABI2, abscisic acid, CRK5, drought tolerance, receptor-like kinase, WRKY18, WRKY40, WRKY60.

## Abstract

Receptor-like kinases (RLKs) have been reported to regulate many developmental and defense process, but only a few members have been functionally characterized. In the present study, our observations suggest that one of the RLKs, a membrane-localized cysteine-rich receptor-like protein kinase, CRK5, is involved in abscisic acid (ABA) signaling in *Arabidopsis thaliana*. Overexpression of *CRK5* increases ABA sensitivity in ABA-induced early seedling growth arrest and promotion of stomatal closure and inhibition of stomatal opening. Interestingly, and importantly, overexpression of *CRK5* enhances plant drought tolerance without affecting plant growth at the mature stages and plant productivity. Transgenic lines overexpressing a mutated form of *CRK5*, *CRK5* ^*K372E*^ with the change of the 372nd conserved amino acid residue from lysine to glutamic acid in its kinase domain, result in wild-type ABA and drought responses, supporting the role of CRK5 in ABA signaling. The loss-of-function mutation of the *CRK5* gene does not affect the ABA response, while overexpression of two homologs of CRK5, CRK4 and CRK19, confers ABA responses, suggesting that these CRK members function redundantly. We further showed that WRKY18, WRKY40 and WRKY60 transcription factors repress the expression of *CRK5*, and that CRK5 likely functions upstream of ABI2 in ABA signaling. These findings help in understanding the complex ABA signaling network.

## Highlight

The cysteine-rich receptor-like protein kinase CRK5 is a potentially positive regulator of ABA signaling in early seedling growth, stomatal movement and plant drought tolerance.

## Introduction

The phytohormone abscisic acid (ABA) plays an essential role in the regulation of plant growth and development, including inhibition of seed germination and seedling growth, promotion of seed dormancy, modulation of stomatal movement, and adaptive responses to various abiotic stresses (Finkelstein *et al.*, 2002). Recent advances in ABA signaling deepen greatly our understanding of the functional mechanism of this phytohormone from primary events of signal perception to downstream gene expression. A group of the START domain proteins PYR/PYL/RCARs, which are identified as cytosolic ABA receptors, interact with protein phosphatase 2Cs (PP2Cs) such as ABA-INSENSITIVE1/2 (ABI1/2) and HYPERSENSITIVE TO ABA1 (HAB1) to release their dephosphorylation effects on SnRK2 protein kinases, which phosphorylate downstream transcription factors to induce ABA-responsive gene expression (Fujii *et al.*, 2009; Ma *et al.*, 2009; Park *et al.*, 2009). The H subunit of Mg-chelatase (CHLH/putative ABA receptor ABAR), which is identified as a candidate receptor for ABA in chloroplasts/plastids, functions together with the chloroplast protein cochaperonin CPN20 and interacts with a group of WRKY transcription factors, WRKY18/40/60, to regulate the expression of downstream ABA-responsive transcription factor genes such as *ABA INSENSITIVE4/5* (*ABI4/ABI5*) (Shen *et al.*, 2006; Wu *et al.*, 2009; Shang *et al.*, 2010; Du *et al.*, 2012; Liu *et al.*, 2012, 2013; Yan *et al.*, 2013; Zhang *et al.*, 2013, 2014). A recent study showed that CHLH/ABAR cross-talks with PYR/PYL/RCARs to regulate SnRK2.6 in stomatal response to ABA (Liang *et al.*, 2015). CHLH/ABAR also regulates a nucleocytosolic PPR-domain protein, SOAR1, which functions as a hub of ABA signaling to nuclear gene expression (Mei *et al.*, 2014; Jiang *et al.*, 2014, 2015; Wang and Zhang, 2014). Functioning at the cell surface, plasma membrane GPCR-type G proteins GTG1 and GTG2 perceive extracellular ABA signals to regulate seed germination and stomatal behavior (Pandey *et al.*, 2009). It is widely believed, however, that ABA signal transduction involves highly complex signaling pathways, and many other components remain to be identified to fully understand the complex ABA signaling network.

Receptor-like kinases (RLKs) have been reported to regulate many developmental and defense process, such as root and shoot growth, cell differentiation regulation, self-incompatibility, brassinosteroid signaling and disease resistance (Morillo & Tax, 2006; De Smet *et al.*, 2009; Osakabe *et al.*, 2013). In *Arabidopsis*, RLKs are the largest membrane receptor family and belong to a large gene family with more than 610 members (Shiu & Bleecker, 2001; Morillo & Tax, 2006). Based on amino acid sequence and structure differences, RLKs are categorized into several subfamilies, including leucine-rich repeat RLKs (LRR-RLKs), cysteine-rich repeat (CRR) RLKs (CRKs), domain of unknown function 26 RLKs, S-domain RLKs, and others (Shiu & Bleecker, 2001). A typical RLK structure contains an extracellular domain, a transmembrane domain and a cytoplasmic kinase domain, whereas receptor-like cytosolic kinases (RLCKs) contain no apparent signal sequence or transmembrane domain (Shiu & Bleecker, 2001; Torii, 2004). Similar to animal receptor tyrosine kinases (RTKs), extracellular domains of RLKs bind to ligand specifically and the conserved intracellular kinase domains transduce signals to their downstream targets in the cytoplasm by catalytic processes of protein phosphorylation (Torii, 2004; Lemmon & Schlessinger, 2010).

Several RLKs have been reported to be involved in ABA signaling pathways and stress tolerance in *Arabidopsis* (Osakabe *et al.*, 2005; Bai *et al.*, 2009; Deng *et al.*, 2009; Xin *et al.*, 2009; Osakabe *et al.*, 2010; Hua *et al.*, 2012; Tanaka *et al.*, 2012; Yu *et al.*, 2012). Mutation of receptor-like kinase 1 (RPK1) decreases ABA sensitivity during the process of seed germination, seedling growth, and stomatal movement, whereas RPK1 overproduction increases plant tolerance to dehydration and oxidative stress (Osakabe *et al.*, 2005; Osakabe *et al.*, 2010). Impairment of proline-rich extensin-like receptor kinase 4 (PERK4) reduces ABA-inhibited root growth by decreasing cytosolic free calcium concentration and Ca^2+^ signaling (Bai *et al.*, 2009). ABA INSENSITIVE3 (ABI3)-activated lectin receptor-like kinase LecRK-b2 positively regulates ABA signaling during seed germination, whereas the A4 subfamily of lectin receptor kinase members, LecRKA4.1, LecRKA4.2 and LecRKA4.3, play negative and redundant roles in ABA responses (Deng *et al.*, 2009; Xin *et al.*, 2009). GUARD CELL HYDROGEN PEROXIDE-RESISTANT1 (GHR1) participates in ABA- and H_2_O_2_-regulated activation of S-type anion currents in guard cells, which can be inhibited by ABI2 but not ABI1 (Hua *et al.*, 2012). A positive regulator of auxin signaling, FERONIA (FER), interacts with guanine exchange factors GEF1, GEF4, and GEF10, which activate GTPase ROP11/ARAC10 and the activated ROP11 enhances the ABI2 phosphatase activity (Yu *et al.*, 2012).

The RLK subfamily of cysteine-rich receptor-like protein kinases (CRKs) includes 46 members in *Arabidopsis*, which are defined by two copies of DUF26 domains each of which contain C–8X–C–2X–C motifs forming disulfide bonds for protein–protein interactions in the extracellular region (Wrzaczek *et al.*, 2010). Over-production of CRK5 or CRK13 enhances plant resistance to *Pseudomonas syringae* (Chen *et al.*, 2003; Acharya *et al.*, 2007). Induced expression of the four structurally closely related CRKs, CRK4, CRK5, CRK19 and CRK20, leads to hypersensitive response-associated cell death in transgenic *Arabidopsis* (Chen *et al.*, 2003, 2004). CRK7 has been reported to be involved in mediating the responses to extracellular ROS production (Idanheimo *et al.*, 2014). The signaling pathways mediated by several CRKs, such as BR-insensitive 1 (BRI1) (Wang *et al.*, 2001) and FLAGELLIN-SENSITIVE2 (FLS2) (Gomez-Gomez *et al.*, 2001), have been well characterized in hormone perception and pathogen response. Two abiotic stress-inducible CRK members, CRK36 and a receptor-like cytosolic kinase (RLCK), ARCK1, interact with each other and negatively regulate ABA and osmotic stress signal transduction (Tanaka *et al.*, 2012). However, the function of most of the CRK members remains unknown.

In this study, we showed that overexpression of a membrane-localized cysteine-rich repeat RLK-encoding gene, *CRK5*, enhances plant sensitivity to ABA and improves drought resistance, whereas overexpression of a mutant form of *CRK5*, *CRK5* ^*K372E*^, induces no significant ABA-related phenotypes. Transgenic lines of the two homologous genes of *CRK5*, *CRK4* and *CRK19*, also exhibit ABA-hypersensitive phenotypes in early seedling growth. Overexpression of *CRK4* also enhances ABA sensitivity of stomatal movement and drought tolerance. The expression of *CRK5* is repressed by cooperation of the WRKY transcription factors WRKY18, WRKY40 and WRKY60. These data suggest that CRK5 is positively involved in ABA signaling. Additionally, genetic evidence suggests that CRK5 may function upstream of ABI2. These findings help in understanding the complex ABA signaling network.

## Materials and methods

### Plant materials and growth conditions

The *Arabidopsis thaliana* ecotype Columbia (Col-0) was used as an Arabidopsis wild-type. The loss of function mutants *crk5-1* (SALK_063519C with Col-0 ecotype as background) and *crk5-2* (SALK_109339 with Col-0 ecotype as background), and the knock-down mutants *crk19-1* (SALK_019639C with Col-0 ecotype as background) and *crk19-2* (SALK_120859C with Col-0 ecotype as background) were purchased from the Arabidopsis Biological Resource Center (ABRC). The seeds of the *aba2* mutant (CS156: *aba2-1*, with Col-0 ecotype as background) were also obtained from ABRC. The *wrky* single, double (*wrky40 wrky18*, *wrky18 wrky60*, and *wrky40 wrky60*), and triple (*wrky40 wrky18 wrky60*) mutants used in this study were identified as described previously (Shang *et al.*, 2010; Liu *et al.*, 2012). The primers for identification of these mutants are listed in Supplementary Table S1 at *JXB* online. For the generation of the *CRK*-overexpression lines, the open reading frame (ORF) sequences of *CRK4*, *CRK5*, *CRK19*, or *CRK20* were amplified by PCR and cloned into the binary vector pCAMBIA-1300-221 (http://www.cambia.org) with a green fluorescent protein (GFP) tag driven by cauliflower mosaic virus (CaMV) 35S promoter. For the generation of the *CRK5* promoter–β-glucuronidase (GUS) transgenic plants, the genomic DNA fragment from 1963bp to 1bp upstream of translation initiation site of *CRK5* was introduced into pCAMBIA-1381 plasmid carrying *GUS* (http://www.cambia.org). The constructed plasmids were introduced into *Agrobacterium tumefaciens* strain GV3101 and then transformed into *Arabidopsis* (Col-0) plants by the floral infiltration method. Transgenic plants with single T-DNA insertion were screened by hygromycin resistance and confirmed by real-time PCR. The homozygous T3 generation seeds were used for further analysis. All the primer sequences used for generation of the transgenic plants are presented in Supplementary Table S1.

The full length sequence of *CRK5* ^*K372E*^, a mutated form of *CRK5* (site-directed mutagenesis of a conserved active-site residue), was obtained by overlap extension PCR (OE-PCR), which introduces a point mutation into the *CRK5* gene sequence by synthesizing a pair of mutated primers (K372E-Middle-F and K372E-Middle-R) that were designed with flanking sequences at the mutation site. Briefly, the procedure of OE-PCR includes two steps. For the first step, the PCR was performed with the wild-type *CRK5* gene as template for amplification of the two mutated *CRK5* ^*K372E*^ segments containing overlapped and mutated sequences using the following primer pairs: forward primer (CRK5-GFP-F) and mutated reverse primer (K372E-Middle-R) for cloning part of the *CRK5* ^*K372E*^ segment with the mutation in its 3′ end; reverse primer (CRK5-GFP-R) and mutated forward primer (K372E-Middle-F) for cloning the other part of the *CRK5* ^*K372E*^ segment with the mutation in its 5′ end. For the second step, the PCR was performed with the two overlapped and mutated *CRK5* ^*K372E*^ segments obtained in the first step as templates for amplification of the full length of the *CRK5* ^*K372E*^ gene using the following primer pair: forward primer (CRK5-GFP-F) and reverse primer (CRK5-GFP-R). The primers used for creating the mutation are listed in Supplementary Table S1. The *CRK5* ^*K372E*^ fragment was cloned into the binary vector pCAMBIA-1300-221 and the methods for transformation of *A. tumefaciens* strain GV3101 and Arabidopsis (Col-0) plants were performed as described above.

For the generation of the *CRK5*/*ABI2* double-overexpression line OE-1×ABI2OE, the over-expressed *ABI2* gene was introduced into the *CRK5*-transgenic line OE-1 by crossing the ABI2OE line with the OE-1 plant. For the generation of the *CRK5*-overexpression plant in the *aba2* mutant background, the *ABA2* gene mutation was introduced into the *CRK5*-transgenic line OE-2 by crossing *aba2* mutant with the OE-2 plant. The precise T-DNA insertion points in OE-1, OE-2 and ABI2OE plants were identified by thermal asymmetric interlaced PCR (TAIL-PCR).

Seeds of different genotypes sown on Murashige and Skoog (MS) medium (Sigma-Aldrich, St Louis, MO, USA) were placed for 72h at 4 °C for stratification and then transferred to a growth chamber at 20–21 °C with about 80 μmol photons m ^−2^ s^−1^ or in compost soil with about 120 μmol photons m ^−2^ s^−1^ light intensity using cool white fluorescent lamps under 16h of light–8h of dark and 60% relative humidity.

### Real-time PCR analysis and TAIL-PCR

The rosette leaves from 4-week-old plants were used for determination of the transcription levels of *CRK5*, *CRK5* ^*K372E*^, *CRK4*, *CRK19* and *CRK20* in the wild-type Col-0 and their corresponding transgenic lines. Seedlings grown 4 days after stratification were sampled for detection of the expression level of ABA-responsive genes. Total RNA was extracted and purified using the total RNA Rapid Extraction Kit (BioTeke, Beijing, China) and RNA Purification Kit (BioTeke, Beijing, China), respectively, according to the manufacturer’s instructions. Single-strand cDNA was synthesized using 2 µg of total RNA with the Roche Transcriptor First Strand cDNA Synthesis Kit (Roche, Mannheim, Germany). Real-time PCR was performed using the CFX96TM Real-Time System of C1000TM Thermal Cycler and SYBR Premix Ex Taq (TaKaRa, Dalian, China) with the program as follows: 5min at 94 °C and then 30 cycles of 5s at 94 °C, 30s at 60 °C. *ACTIN2/8* gene was used as an internal control. All the real-time PCR assays were performed in triplicate and means of the three biological repeats were calculated to represent gene expression level. Primers for real-time PCR are listed in Supplementary Table S1. TAIL-PCR was performed essentially as described previously (Liu *et al.*, 1995; Mei *et al.*, 2014). Random primers and the specific left border primer of pCAMBIA-1300-221 are listed in Supplementary Table S1.

### Phenotypic analysis

For the cotyledon greening assay, about 200 seeds of different genotypes were sterilized and planted on ABA-free or (±)ABA-containing MS medium that contained 3% sucrose and 0.7% agar (pH 5.8~6.0). The seeds were placed at a growth chamber after stratification for 72h, and green cotyledons were scored 5 days later.

Two methods were used to test ABA-mediated inhibition of post-germination growth. For the first method, the seeds were directly planted in ABA-free or ABA-containing MS medium, and seedling growth was recorded at the indicated times when the primary root length was measured using a ruler. For the second method, seeds were planted in ABA-free medium, subjected to a 3-d stratification, and 60-h-old germinating seeds/young seedlings were transferred to ABA-free (0 μM) or (±)ABA-containing MS medium and continued to grow for 10 d before investigation.

Stomatal aperture was determined essentially as described previously (Wu *et al.*, 2009; Shang *et al.*, 2010). Rosette leaves of 4-week-old plants were used. To assay ABA-induced stomatal closure, detached leaves of different genotype plants were immersed in solutions containing 50mM KCl and 10mM MES-KOH (pH 6.15) under a halogen cold light source (Colo-Parmer) for 3h before treatment with different concentrations of (±)ABA for 2h. Apertures were recorded on epidermal strips to estimate ABA-induced stomatal closure before and after ABA treatment. To assay ABA-inhibited stomatal opening, plants were placed in dark for 24h before leaves were immersed in the same buffer described above containing different concentration of ABA for 2h under the cold light, and the apertures were then determined.

For drought stress treatment, plants of different genotypes grown for 2 weeks on ABA-free MS medium under normal conditions were transferred into soil and placed in the greenhouse without irrigation. After 16 d of water deficiency, the plants’ growth status was photographed before and after re-watering for 24h followed by recording and calculating survival rates.

### Yeast one-hybrid assays

Yeast one-hybrid assays were performed with a Matchmaker™ One-Hybrid Library Construction and Screening kit (Clontech, Mountain View, CA, USA) using the AH109 yeast strain according to the manufacturer’s instructions. The promoter fragment of *CRK5* and the open reading frame of *WRKY18/40/60* were cloned into the pHIS2 bait vector and pGADT7 prey vector, respectively. Yeast cells were co-transformed with pGADT7 prey vector containing *WRKY18*, *WRKY40*, or *WRKY60* and pHIS2 bait vector containing the promoter of *CRK5*. The corresponding transformation with pGADT7 prey vector containing *WRKY18*, *WRKY40*, or *WRKY60* and pHIS2 bait vector containing *p53* promoter fragment were used as negative controls. Co-transformation of pHIS2-*p53* with pGADT7-*p53* was used as positive control, and co-transformation of pGADT7-*p53* with empty pHIS2 was used as its own negative control. Transformed yeast cells with different combinations of plasmids were first grown in SD-2 medium (lacking Trp, Leu) at 30 °C for 4 days to ensure that the yeast cells were successfully co-transformed, and then co-transformed yeast cells were grown overnight in liquid SD-2 medium to an OD_600_ of 0.1 and diluted in a 10× dilution series. For each dilution, 10 μl yeast cells was spotted on SD-2 and SD-3 medium (lacking Trp, Leu, His) supplemented with 40mM 3-aminotriazole (3-AT; Sigma-Aldrich, USA) and then cultured at 30 °C for another 4 days. The primers used for constructing the related plasmids are listed in Supplementary Table S1.

### *Trans*-inhibition of *CRK5* promoter activity by WRKYs in tobacco leaves

This experiment was performed essentially according to the previously described procedures (Liu *et al.*, 2012). The full-length ORF fragments of the *WRKY* genes were amplified by PCR and cloned into the pCAMBIA1300-Flag vectors under the promotion of CaMV 35S promoter, forming the effector constructs. The *CRK5* promoter fused with the reporter construct, a modified form of pCAMBIA-1381 vector with the full length ORF of luciferase (LUC) gene, was inserted into the Sal1/Spe1 sites before the start codon of the GUS reporter gene. Primers used are listed in Supplementary Table S1. The constructs were transformed into *A. tumefaciens* strain GV3101. Bacterial suspensions were infiltrated into healthy and fully expanded leaves of 7-week-old *Nicotiana benthamiana* plants using a needleless syringe. The amounts of the infiltrated constructs must be kept the same among treatments and controls for each group of assays. After infiltration, plants were placed in darkness for 24h and then with 16h of light–8h of dark for another 24h, and the LUC activity (assessed by fluorescence intensity) was observed 48h after infiltration with CCD imaging apparatus (Andor iXon, Andor, UK). We used the ImageJ software (an image processing program developed by the National Institutes of Health, which can calculate area and pixel value statistics of user-defined areas and intensity-thresholded objects) to calculate the average optical density (OD, integrated density divided by area) of the fluorescence area and background area of the experimental groups and control groups, respectively. The values of the fluorescence intensity as shown in Fig. 10C–E are the result of the OD value of fluorescence area minus the corresponding OD value of background area.

### Protein production and purification in *E. coli*

6×His-tagged full length proteins of WRKY18, WRKY40 and WRKY60 were produced in *E. coli* and purified essentially as described previously (Wu *et al.*, 2009; Shang *et al.*, 2010). The cDNA fragments encoding these proteins were cloned by PCR and the primers are listed in Supplementary Table S1. Full-length ORFs of *WRKY18*, *WRKY40* and *WRKY60* were cloned into the protein expression vector pET48b(+). The recombinant plasmids were transformed and expressed in *E. coli* BL21 (DE3) strains (Novagen, Darmstadt, Germany). The transformed *E. coli* were grown at 37 °C overnight in 1 liter of liquid Luria–Bertani (LB) medium containing 50 μg ml^–1^ kanamycin until the OD_600_ of the cultures was 0.6–0.8. Then, isopropyl-β-D-thiogalactopyranoside was added to the cultures to a final concentration of 0.5mM, and culture was continued at 16 °C at 150rpm for 16h. The WRKY18, WRKY40 and WRKY60 proteins were expressed in the inclusion body, and resolved by 8M urea after the collected cells were lysed, followed by protein purification using a Ni^2+^-chelating column (Novagen, San Diego, CA USA) as described in the manufacturer’s instructions for the Ni^2+^-chelating column. After that, the denatured WRKY proteins were treated with slow dialysis in a dialysis buffer containing 25mM Tris (pH 8.0), 150mM NaCl, and 1×Protease Inhibitor Cocktail (Roche, Mannheim, Germany) with a gradually decreased amount of urea (6, 4, 2, 0M) for about 24h until renaturation of recombinant proteins. Sodium dodecyl sulfate (SDS)-polyacrylamide gel electrophoresis (PAGE) was then conducted to detect the quality of purified proteins.

### Gel shift assay

Gel shift assay (GSA) was performed with a Light-Shift Chemiluminescent EMSA Kit (Thermo Scientific, Waltham, MA, USA) using the recombinant 6×His-WRKY18, 6×His-WRKY40 and 6×His-WRKY60 fusion proteins purified from *E. coli* according to the manufacturer’s instructions. The promoter fragments used for the GSA were synthesized using the following primer pairs: forward primer 5′-TTGATGTTACTCGTCTAGTTGACCTTGACTTGCAAG ATATTGTATTATTTTACAAAAACCAAAATTTGACT GGCTTGGCT-3′ and reverse primer 5′-AGCCAAGCCAGTCAAA TTTTGGTTTTTGTAAAATAATACAATATCTTGCAAGTC AAGGTCAACTAGACGAGTAACATCAA-3′ for the *CRK5* promoter fragment *ProCRK5-1*; forward primer 5′-TTGATGTTACTCGTCTAGTTGACCTTGACTTGCAA GATATTGTATTATTTTACAAAAACCAAAATTT AAATGGCTTGGCT-3′ and reverse primer 5′-AGCCAAGCCATT TAAATTTTGGTTTTTGTAAAATAATACAA TATCTTGCAAGTCAAGGTCAACTAGACG AGTAACATCAA-3′ for the W-box mutation of W1 in the fragment *ProCRK5-1*; forward primer 5′-TTGATGTTACTCGTCTA GTTAAACTTAAATTGCAAGATATTGTATTATT TTACAAAAACCAAAATTTGACTGGCTTG GCT-3′ and reverse primer 5′-AGCCAAGCCAGTCAAATT TTGGTTTTTGTAAAATAATACAATATCTTGCAATTTAA GTTTAACTAGACGAGTAACATCAA-3′ for the double W-box mutations of W2 and W3 in the fragment *ProCRK5-1*. The fragment of *ProCRK5-1* and its mutant form mW1 and mW2/W3 were synthesized directly by annealing of the above described forward primers and reverse primers, and each of the primers were synthesized with the biotin labeling in the 5′ end for biotin-labeled fragment or synthesized without labeling for competitor fragments. Forward primer 5′-AGTTGTAAAGTTCAGAAGGAAAAGTACTAA-3′ and reverse primer 5′-GGATATTTAATAGGTTTGTGATTATTCAG-3′ were used for PCR amplification of the fragment *ProCRK5-2*. Forward primer 5′-AGTATAAGATGGGTTGTGGTGACTATAAGA-3′ and reverse primer 5′-TGGAAGTAATTTAACTAAGAA AAATCGAAG-3′ were used for PCR amplification of the fragment *ProCRK5-3*. Biotin labeled fragments were obtained by PCR amplification using the above described primer pairs with the first base in the 5′ end labeled with biotin. Unlabeled fragments of the same sequences were used as competitors. Binding reactions were performed in a binding buffer containing 25mM Hepes (pH 8.0), 40mM KCl, 5mM MgCl_2_, 1mM DTT, 1mM EDTA and 8% glycerol with 50ng recombinant 6×His-WRKY fusion protein and 20fmol probes for each of the biotin-labelled promoter fragments. Competition binding experiments were performed using a 50- and 200-fold molar excess of unlabeled fragments and the mixture was cultured for 30min at 28 °C followed by PAGE without SDS.

### Protein targeting and histochemical analysis

Roots of 1-week-old transgenic plants expressing *CRK5-GFP* or empty *GFP* driven by CaMV 35S promoter were used to detect the subcellular localization of CRK5 or GFP, respectively, using a confocal laser scanning microscope (LSM780, Carl Zeiss, Germany). The chemical reagent *N*-(3-triethylammomiumpropyl)-4-(*p*-diethylaminophenylhexatrienyl) (FM4-64; Invitrogen, Carlsbad, CA, USA) is widely used as an endocytic tracer and plasma membrane stain that offers red fluorescence (Fischer-Parton *et al.*, 2000; Lu *et al.*, 2016). For the FM4-64 staining, roots were immersed in 20ng ml^–1^ FM4-64 solution for 2min before investigation under a confocal microscope. GFP fluorescence was detected using an emission filter at 505–530nm with excitation at 488nm, and the red signal of FM4-64 staining was collected using an emission filter at 585–615nm with excitation at 543nm.

For the GUS staining, whole plants or tissues of the transgenic lines expressing *CRK5*-promoter-GUS were immersed in a reaction buffer containing 1mM 5-bromo-4-chloro-3-indolyl-β-GlcUA (X-gluc; Sigma-Aldrich, USA), 100mM sodium phosphate (pH 7.0), 2mM EDTA, 0.05mM ferricyanide, 0.05mM ferrocyanide and 0.1% (v/v) Triton X-100 for 12h at 37 °C. Chlorophyll was removed from the tissues with a mixture of 30% acetic acid and 70% ethanol.

## Results

### Overexpression of *CRK5*, but not its mutated form *CRK5* ^*K372E*^, results in ABA hypersensitivity in post-germination growth

Our preliminary experiment suggested that expression of *CRK5* is likely to be regulated by the ABA-responsive WRKY transcription factors WRKY18/40/60, which are negatively involved in ABA signaling (Shang *et al.*, 2010; Liu *et al.*, 2012, 2013; Yan *et al.*, 2013; Zhang *et al.*, 2013, 2014). To test whether *CRK5* is involved in ABA signaling, we created transgenic plants overexpressing *CRK5* or its mutated form *CRK5* ^*K372E*^. It is known that the mutation of the conserved lysine to glutamic acid abolishes the activities and functions of RLKs (Chen *et al.*, 2003, 2004; Torii, 2004; Wang *et al.*, 2008; Lemmon and Schlessinger, 2010; Osakabe *et al.*, 2010; Tanaka *et al.*, 2012). An amino acid sequence alignment of the conserved cytoplasmic kinase domain of the *Arabidopsis* receptor-like protein kinases CRK5, CRK36, ARCK1, BAK1 and RPK1 indicated that the 372nd amino acid is the conserved lysine in the kinase domain of CRK5 protein (see Supplementary Fig. S1), and thus the site of the conserved lysine 372 was chosen for point mutagenesis. The CRK5^K372E^ mutation, which involves the change of the 372nd lysine to glutamic acid residue in the kinase domain of CRK5, may lead to loss-of-function of this protein kinase.

**Fig. 1.**
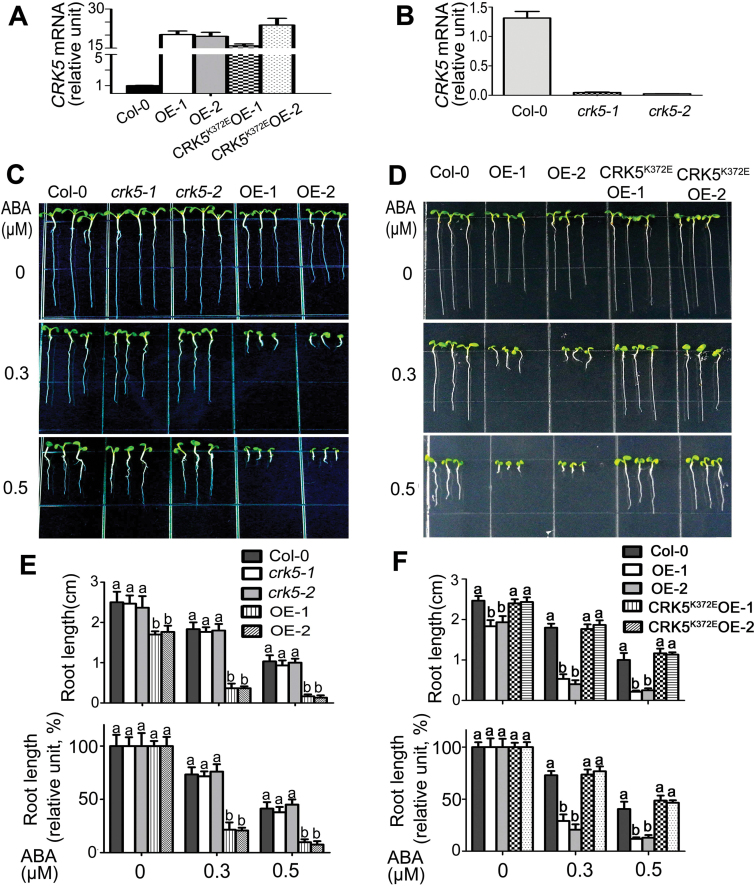
Overexpression of *CRK5*, but not its mutated form *CRK5* ^*K372E*^, results in an ABA-hypersensitive phenotype in early seedling growth. (A) Real-time PCR analysis of the transgenic lines overexpressing *CRK5* (OE-1 and OE-2) or a mutated form of *CRK5* encoding CRK5^K372E^ with a point mutation at its kinase domain (CRK5^K372E^ OE-1 and OE-2). Expression level of *CRK5* or *CRK5* ^K372E^ was normalized to that of *Actin2/8*, and the expression level of *CRK5* in Col-0 was set to 1. Values are the mean±SE of three independent biological determinations, and different letters represent significant differences at *P*<0.05 (Duncan’s multiple range test). (B) Real-time PCR analysis of the *CRK5* expression level in wild-type Col-0, and *crk5-1* and *crk5-2* T-DNA insertion mutant plants. Values are the mean±SE of three independent biological determinations, and different letters represent significant differences at *P*<0.05 (Duncan’s multiple range test). (C, D) Root growth of wild-type Col-0, *crk5-1*, *crk5-2*, OE-1, OE-2 (C) or Col-0, OE-1, OE-2, CRK5^K372E^OE-1 and CRK5^K372E^OE-2 (D) growing on ABA-free (0 μM) or (±)ABA-containing (0.3 and 0.5 μM) MS medium. Seeds were directly planted in the medium for a 72-h stratification and germinating seeds/young seedlings continued to grow for 10 d before investigation. The experiments were repeated three times with similar results. (E, F) Statistical analysis of absolute (top) and relative values (bottom) of root length of different genotypes described in (C) and (D), respectively. Relative values of the root length of each genotype grown on MS medium containing 0.3 and 0.5 μM (±)ABA are normalized relative to the value of the corresponding genotype at 0 μΜ (±)ABA, which is taken as 100%. Values are the mean±SE of three biological determinations, and different letters represent significant differences at *P*<0.05 (Duncan’s multiple range test).

**Fig. 2.**
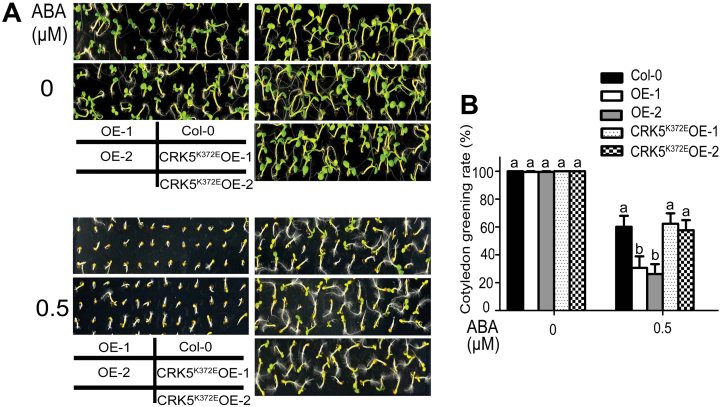
Overexpression of *CRK5*, but not its mutated form *CRK5* ^*K372E*^, results in an ABA-hypersensitive phenotype in ABA-induced inhibition of cotyledon greening. (A) Cotyledon greening of wild-type Col-0, *CRK5*-transgenic lines OE-1 and OE-2, and *CRK5* ^*K372E*^-transgenic lines CRK5^K372E^OE-1 and CRK5^K372E^OE-2 in ABA-free (0 μM, top) or (±)ABA-containing (0.5 μM, bottom) MS medium. (B) Percentages of green cotyledons of the different genotypes as described in (A). Green cotyledons were scored 5 days after stratification. Values are the mean±SE of three biological determinations, and different letters represent significant differences at *P*<0.05 (Duncan’s multiple range test).

**Fig. 3.**
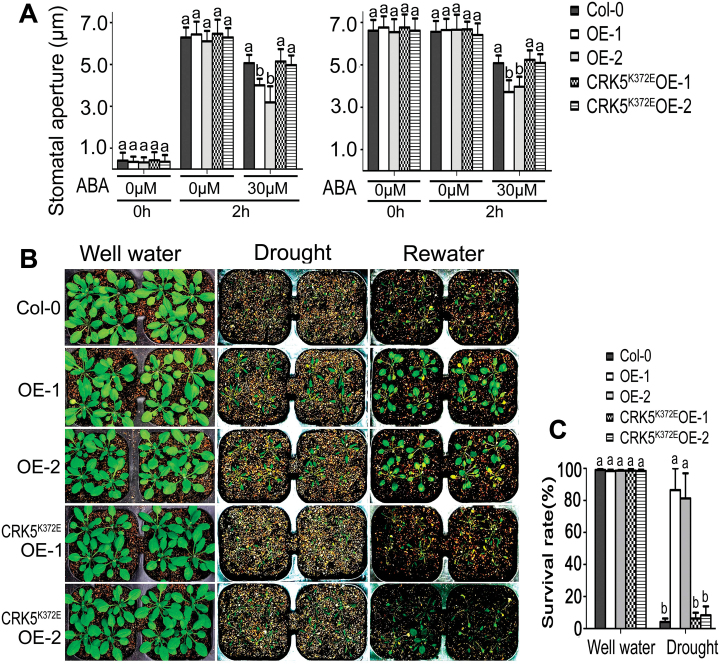
Overexpression of *CRK5*, but not *CRK5* ^*K372E*^, results in ABA hypersensitivity in stomatal movement and increases plant drought tolerance. (A) ABA-induced inhibition of stomatal opening (left) and promotion of stomatal closure (right) in wild-type Col-0 plants, *CRK5*-transgenic lines OE-1 and OE-2, and *CRK5* ^*K372E*^-transgenic lines CRK5^K372E^OE-1 and CRK5^K372E^OE-2. The experiments were repeated five times with similar results. The values are the mean±SE from 60 stomata for each time point, and different letters represent significant differences at *P*<0.05 (Duncan’s multiple range test). (B) Test of drought tolerance of the different genotypes described above. Plants were well watered (control, ‘Well water’) or drought stressed by withholding water (‘Drought’) for 16 d (D) and then re-watered (‘Rewater’). The experiments were repeated five times, and at least 30 plants per individual line were used for each experiment. (C) Survival rates of the plants described in (B). The values are the mean±SE of three biological determinations, and different letters represent significant differences at *P*<0.05 (Duncan’s multiple range test).

**Fig. 4.**
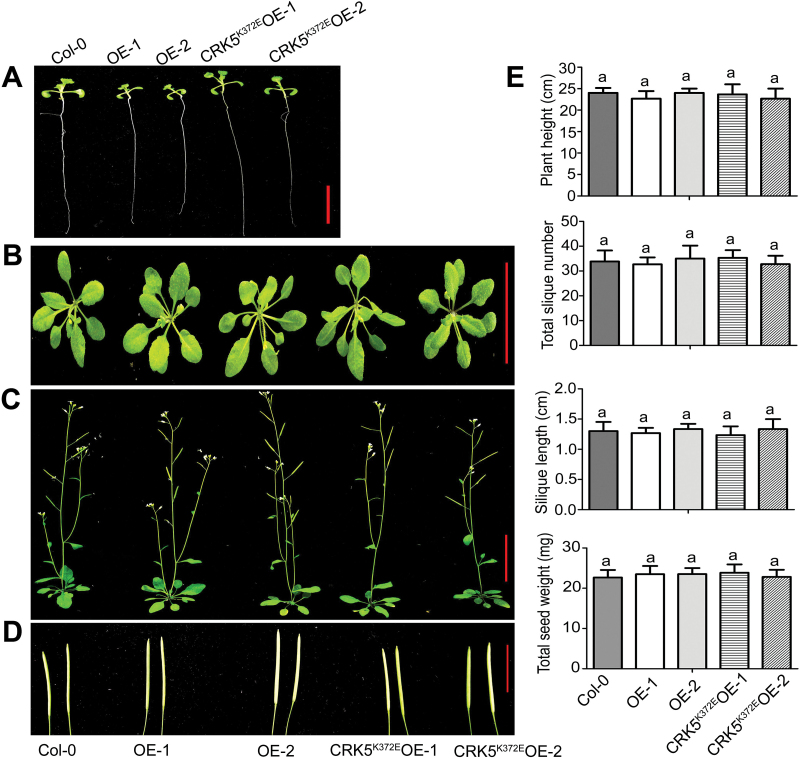
Effects of over-expression of *CRK5* on plant growth and productivity. Two-week-old (A; bar=1cm), 4-week-old (B; bar=3cm), and 6-week-old seedlings (C; bar=4cm), and siliques (D; bar=1cm) are shown for wild-type Col-0 plants, *CRK5*-transgenic lines OE-1 and OE-2, and *CRK5* ^*K372E*^-transgenic lines CRK5^K372E^OE-1 and CRK5^K372E^OE-2. (E) Statistics of the plant height, total silique number per plant, silique length and total seed weight (dry weight) per plant of the different genotypes as described above. Each value is the mean±SE of three biological determinations, and different letters represent significant differences at *P*<0.05 (Duncan’s multiple range test).

**Fig. 5.**
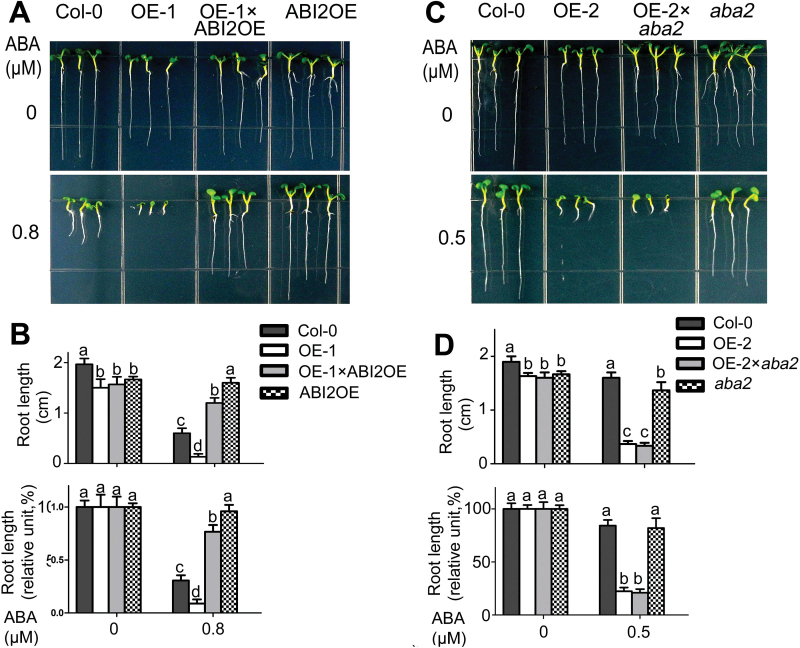
Test of genetic interaction of CRK5 with ABI2 involved in ABA signaling or with ABA2 involved in ABA biosynthesis. (A) ABI2 is genetically epistatic to CRK5. Seeds of wild-type Col-0, *CRK5*-overexpression line OE-1, *ABI2*-overexpression line ABI2OE and *CRK5*/*ABI2*-double-overexpression line OE1×ABI2OE were directly planted on the ABA-free (0 μM) or 0.8 μM-ABA-containing MS medium, and the growth status was recorded 10 d after stratification. The experiments were repeated three times with similar results. (B) Statistical analysis of absolute (top) and relative values (bottom) of root length of different genotypes described in (A). Relative values of the root length of each genotype grown on ABA-containing medium are normalized relative to the value of the corresponding genotype at 0 μM ABA, which is taken as 100%. Values are the mean±SE of three biological determinations, and different letters represent significant differences at *P*<0.05 (Duncan’s multiple range test). (C) Loss-of-function of ABA2 (*aba2*) does not affect ABA-hypersensitive response of the *CRK5*-overexpression line OE-2. Seeds of wild-type Col-0, *aba2* mutant, *CRK5*-overexpression line OE-2 and *CRK5*-overexpression line OE2 in the *aba2* mutant background (OE-2×*aba2*) were directly planted on the ABA-free (0 μM) or 0.5 μM-ABA-containing MS medium, and the growth status was recorded 10 d after stratification. The experiments were repeated three times with similar results. (D) Statistical analysis of absolute (top) and relative values (bottom) of root length of different genotypes described in (C). Relative values of the root length of each genotype grown on ABA-containing medium are normalized relative to the value of the corresponding genotype at 0 μM ABA, which is taken as 100%. Values are the mean±SE of three biological determinations, and different letters represent significant differences at *P*<0.05 (Duncan’s multiple range test).

We selected five *CRK5*-overexpression lines (OE-1, OE-2, OE-5, OE-6 and OE-7) and five *CRK5* ^*K372E*^-overexpression lines (CRK5^K372E^OE-1, CRK5^K372E^OE-2, CRK5^K372E^OE-3, CRK5^K372E^OE-5 and CRK5^K372E^OE-7) as representatives used in this study (Figs 1–5; Supplementary Figs S2 and S3). Two loss-of-function T-DNA insertion mutant alleles of the *CRK5* gene, *crk5-1* (Salk_063519C) and *crk5-2* (SALK_109339), were also obtained and identified (Fig. 1B). We assayed ABA sensitivity of the different genotypes by directly sowing seeds in ABA-free or ABA-containing medium, and observed that the *CRK5*-overexpression lines OE-1 and OE-2 were significantly hypersensitive to ABA in ABA-induced post-germination growth arrest, estimated by both root length and cotyledon greening rate (Figs 1C–F and 2A, B). The *GFP*-transgenic plants showed wild-type ABA response (see Supplementary Fig. S4), excluding the possible disturbance of the GFP tag of CRK5 in the experiments. It is noteworthy that the *CRK5*-transgenic lines OE-1 and OE-2 exhibited shorter roots compared with wild-type plants in ABA-free medium, which may be due to a complex, currently unknown, function of CRK5 to reduce root growth, while the root length of the OE-1 and OE-2 lines was significantly much more reduced than that of wild-type plants in the presence of ABA treatment (Fig. 1E, F). However, the loss-of-function mutants *crk5-1*and *crk5-2*, as well as *CRK5* ^*K372E*^-overexpression lines CRK5^K372E^OE-1 and CRK5^K372E^OE-2, showed wild-type ABA responses in ABA-induced post-germination growth arrest (Figs 1C–F and 2A, B).

We used a different technique to further test the ABA response of early seedling growth of these different genotypes. Seeds were planted in ABA-free medium, subjected to a 3-d stratification, and 60-h-old germinating seeds/young seedlings were transferred to ABA-containing medium and continued to grow for 10 d before investigation. The same ABA-hypersensitive phenotypes of the *CRK5*-overexpression lines OE-1 and OE-2 were observed, while other genotypes, including *crk5-1*, *crk5-2*, CRK5^K372E^OE-1 and CRK5^K372E^OE-2, showed wild-type ABA responses (see Supplementary Fig. S2). These findings confirmed the observations mentioned above. Together, these findings suggest that CRK5 is positively involved in ABA signaling as a functional protein kinase and that a functional redundancy occurs in the CRK-mediated ABA signaling.

Moreover, we observed that the ABA sensitivity of the different *CRK5*-transgenic lines OE-1, OE-5, OE-6 and OE-7 with a gradient of the *CRK5* expression levels was positively correlated with the *CRK5* expression levels (see Supplementary Fig. S3A, C, E), whereas the *CRK5* ^*K372E*^ expression did not modify the ABA response regardless of its expression levels (Supplementary Fig. S3B, D, F). These findings further suggest that CRK5 is positively involved in ABA signaling.

### Overexpression of *CRK5*, but not *CRK5* ^*K372E*^, results in ABA hypersensitivity in stomatal movement and increases plant drought tolerance without affecting plant productivity under non-stressful conditions

The *CRK5*-overexpression lines OE-1 and OE-2 showed significant ABA-hypersensitive phenotypes, whereas the *CRK5* ^*K372E*^-transgenic lines CRK5^K372E^OE-1 and CRK5^K372E^OE-2 exhibited wild-type ABA responses, in ABA-induced promotion of stomatal closure and inhibition of stomatal opening (Fig. 3A). We further assayed dehydration tolerance of these genotypes, and observed that the *CRK5*-overexpression lines OE-1 and OE-2 showed significantly higher tolerance to drought than the *CRK5* ^*K372E*^-transgenic lines CRK5^K372E^OE-1 and CRK5^K372E^OE-2 that exhibited drought-sensitive as wild-type plants (Fig. 3B, C). These findings are consistent with the ABA-hypersensitive phenotypes of the *CRK5*-overexpression lines in stomatal movement.

As plant drought tolerance is often associated with reduction of plant development, and the *CRK5*-transgenic plants displayed shorter roots compared with the wild-type plants in the early growth stage, we assayed the plant growth and productivity of wild-type Col-0, the *CRK5*-overexpression lines OE-1 and OE-2, and the *CRK5* ^*K372E*^-transgenic lines CRK5^K372E^OE-1 and CRK5^K372E^OE-2 at the different mature stages under non-stressful conditions. We found no significant differences in the aspects of rosette leaves, plant height, total silique number per plant, silique length and total seed weight (dry weight) per plant among the different genotypes (Fig. 4A–E).

### Functional interaction of CRK5 with ABI2 and ABA2

We created *CRK5*/*ABI2*-double-overexpression line OE-1×ABI2OE line (Fig. 5; Supplementary Fig. S5) and the *CRK5*-overexpressing line OE-2 in the *aba2* mutant background (OE-2×*aba2*; Fig. 5; Supplementary Fig. S5). The OE-1×ABI2OE line showed an ABA-hyposensitive phenotype in early seedling growth like the *ABI2*-overexpression line ABI2OE, which suppresses the ABA-hypersensitive phenotype of the *CRK5*-overexpressing line OE-1 (Fig. 5A, B). These findings reveal that ABI2 is genetically epistatic to CRK5, suggesting that CRK5 may function upstream of ABI2 in ABA signaling. To the contrary, the OE-2×*aba2* plants displayed an ABA-hypersensitive phenotype in early seedling growth like the *CRK5*-overexpression line OE-2, so loss-of-function of ABA2 (*aba2*) did not affect the ABA-hypersensitive response resulting from overexpression of *CRK5* (Fig. 5C, D). Given that ABA2 is a key, rate-limiting enzyme for ABA biosynthesis (Nambara & Marion-Poll, 2005; Taylor *et al.*, 2005), these data reveal that CRK5 regulates ABA signaling independently of ABA biosynthesis. However, we further found that *CRK5*-overexpression could partly restored the drought-sensitive phenotype of the *aba2* mutant (see Supplementary Fig. S6A, B), suggesting that overexpression of CRK5 stimulates drought response of this mutant by promoting cell signaling in response to the low level of ABA in the *aba2* mutant.

### CRK5 protein is localized to plasma membrane, and *CRK5* gene is expressed preferentially in roots and leaves

**Fig. 6.**
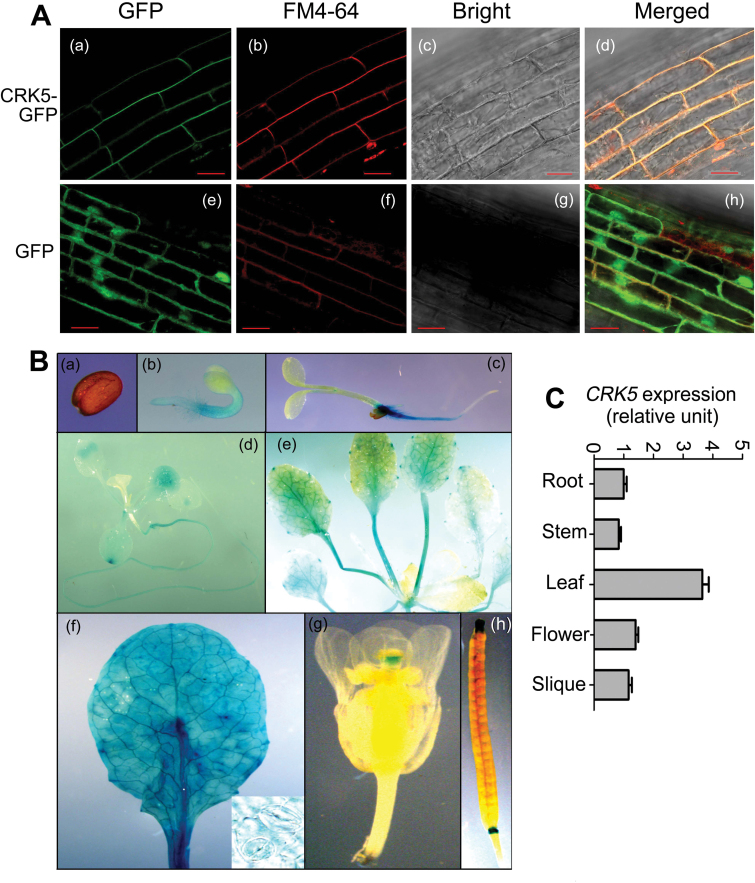
Subcellular localization of CRK5 protein and expression profile of *CRK5* gene. (A) CRK5 is localized to plasma membrane. The Col-0 plants were transformed with the construct carrying *CRK5-GFP* or empty *GFP*, respectively, driven by CaMV 35S promoter, and the roots of transgenic plants were investigated by a confocal laser scanning microscope. (a) CRK5–GFP localization in the mature root zone. (b) FM4-64 staining of the *CRK5-GFP*-transgenic plant in the mature root zone. (c) The corresponding bright field of (a) and (b). (d) Merged imagine of (a), (b) and (c). (e) Empty GFP localization in the mature root zone. (f) FM4-64 staining of *GFP*-transgenic plants in the mature root zone. (g) The corresponding bright field (e) and (f). (h) Merged imagine of (e), (f) and (g). Bars=20 μm. (B) Expression of the *CRK5*-promoter–*GUS* in transgenic lines. (a) Dry seed. (b) Young seedling 48h after stratification. (c) Young seedling 72h after stratification. (d) Young seedling 14 d after stratification. (e) Young seedling 21 d after stratification. (f) Rosette leaves and stomata (shown at bottom, right). (g) Flower. (h) Silique. (C) Relative expression levels of *CRK5* in different tissues/organs determined by real-time PCR analysis.

We used the homozygous transgenic plants expressing the CRK5 protein fused with GFP (CRK5–GFP) to investigate the subcellular localization of CRK5. Confocal imaging showed that CRK5–GFP fusion protein was localized in the plasma membrane of the roots of the transgenic plants (Fig. 6A), and that the GFP fluorescence of CRK5–GFP protein merged well with the red fluorescence of the FM4-64 dye that stains the plasma membrane (Fig. 6A). It is noteworthy that FM4-64 is a lipophilic probe used as an endocytic tracer to study the vesicle trafficking of the plasma membrane, and so can be used as a transient plasma membrane stain (within 10min after staining) (Fischer-Parton *et al.*, 2000; Lu *et al.*, 2016). In this experiment, we observed that the fluorescence of FM4-64 was slightly moved from the plasma membrane to cytoplasmic space (Fig. 6A, b, bottom), which may be a phenomenon of endocytosis. As a control, the GFP fluorescence of transgenic plants expressing GFP protein alone were found in the nucleus, cytoplasm and membranes, and only the green signal in the membrane merged with the red fluorescence of FM4-64 (Fig. 6A). Consistently, a prediction with the ‘DAS’ Transmembrane Prediction server and TMHMM algorithm suggests that CRK5 is associated with cell membranes (see Supplementary Fig. S7). Together, these data showed that CRK5 is a plasma membrane-localized protein.

We created the *CRK5*-promoter–*GUS* transgenic lines to investigate the spatial expression pattern of *CRK5*, and observed that *CRK5* was ubiquitously expressed in all the organs/tissues except for seeds (Fig. 6B). The GUS-expression level appeared higher in roots and leaves, but almost no GUS staining was detected in the seeds, including dry seeds, imbibed seeds and mature seeds residing in siliques (Fig. 6B). Similarly, the real-time PCR data showed that the *CRK5* gene was expressed in different organs/tissues and had a higher expression level in leaves than in other tissues (Fig. 6C).

### Overexpression of *CRK5*, but not its mutated form *CRK5* ^*K372E*^, alters expression of a subset of ABA-responsive or ABA-signaling-related genes

**Fig. 7.**
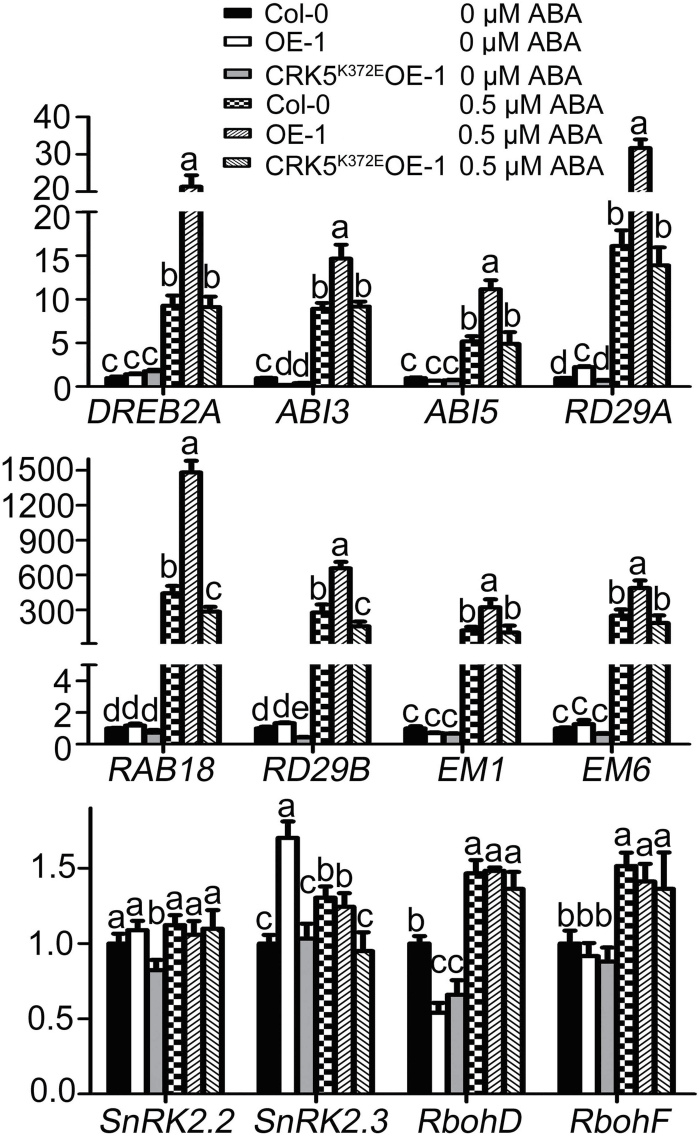
Expression of some ABA-responsive genes in *CRK5*-transgenic line OE-1 and *CRK5* ^*K372E*^-transgenic line CRK5^K372E^OE-1. The seeds were germinated and grown on ABA-free (–ABA) or 0.5 μM-ABA-containing (+ABA) MS medium for 4 days before sampling for RNA extraction. Transcription levels of these genes were assayed by real-time PCR. Expression level of each gene is normalized to that of *Actin2/8*, and the relative expression level of each gene is normalized relative to the level of this gene of the wild-type Col grown in ABA-free medium, which is taken as 1. Values are the mean±SE of three independent biological determinations, and different letters represent significant differences at *P*<0.05 (Duncan’s multiple range test).

We assayed the expression levels of a subset of ABA-responsive or ABA-signaling-related genes in *CRK5* transgenic line OE-1 and *CRK5* ^*K372E*^-transgenic line CRK5^K372E^OE-1. The assayed ABA-responsive or ABA-signaling-related genes include *RD29A* and *RD29B* (Yamaguchi-Shinozaki & Shinozaki, 1994), *RAB18* (Lang & Palva, 1992), *DREB2A* (Liu *et al.*, 1998), *ABI3* (Giraudat *et al.*, 1992), *ABI5* (Finkelstein & Lynch, 2000), *EM1* and *EM6* (Gaubier *et al.*, 1993; Devic *et al.*, 1996), *SnRK2.2, SnRK2.3* (Fujii & Zhu, 2009), *RbohD* and *RbohF* (Kwak *et al.*, 2003). In the absence of ABA, expression levels of these genes in Col-0, OE-1 and CRK5^K372E^OE-1 showed no marked difference except *SnRK2.3* with a higher level in OE-1 and *RbohD* with a lower level in both OE-1 and CRK5^K372E^OE-1 (Fig. 7). In the presence of ABA, the expression levels of *RD29A*, *RD29B*, *RAB18*, *DREB2A*, *ABI3*, *ABI5*, *EM1* and *EM6* were significantly and markedly increased in the *CRK5* overexpression line, whereas the expression levels of these genes in the CRK5^K372E^OE-1 line were not altered significantly except for *RAB18* and *RD29B* of which the expression slightly decreased in the CRK5^K372E^OE-1 line (Fig. 7). No significant difference was detected for the expression of other ABA-signaling regulator-encoding genes including *SnRK2.2*, *RbohD* and *RbohF* in the OE-1 and the CRK5^K372E^OE-1 lines compared with the wild-type plants in the presence of ABA (Fig. 7). These data of the expression of the ABA-responsive genes are essentially consistent with the ABA-related phenotypes of the *CRK5* transgenic lines and wild-type responses of the *CRK5* ^*K372E*^-transgenic lines.

### Overexpression of homologs of *CRK5*, *CRK4* and *CRK19*, but not *CRK20*, results in ABA hypersensitivity in post-germination growth

**Fig. 8.**
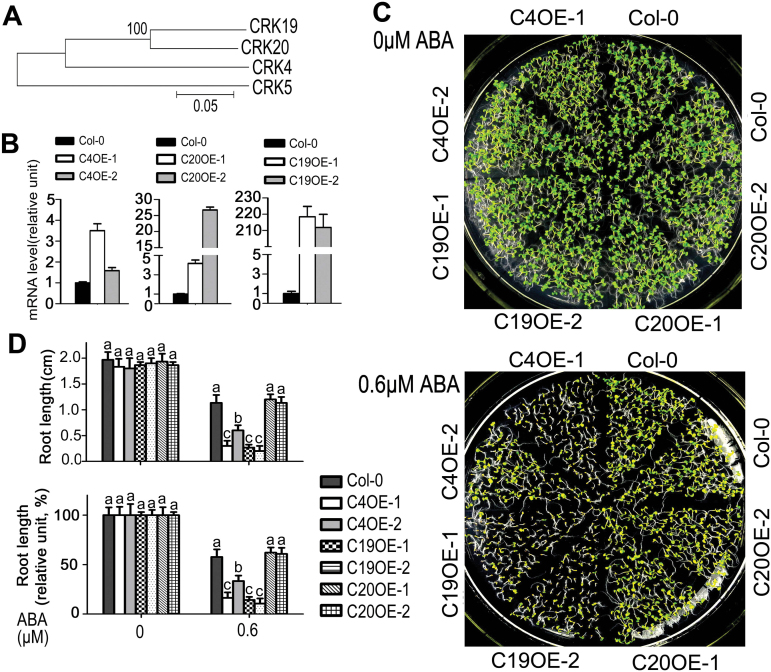
Phenotypes of the transgenic lines overexpressing *CRK4*, *CRK19* or *CRK20* homologous to *CRK5* in ABA-induced early seedling growth arrest. (A) Phylogenic analysis of *Arabidopsis* CRK4, CRK5, CRK19 and CRK20 using the neighbor-joining method with MEGA version 4 by alignment of the amino acid sequences with ClustalW. (B) Real-time PCR analysis of the transgenic lines. C4OE-1 and C4OE-2 denotes *CRK4*-overexpression lines; C19OE-1 and C19OE-2, *CRK19*-overexpression lines; C20OE-1 and C20OE-2, *CRK20*-overexpression lines. Values are the mean±SE of three independent biological determinations. (C) Phenotypes of ABA-induced inhibition of early seedling growth in different transgenic lines as described in (B). Seeds were directly planted in ABA-free (0 μM ABA, top) or 0.6 μM-ABA-containing MS medium, and the growth status was recorded 10 d after stratification. The experiments were repeated three times with similar results. (D) Statistical analysis of absolute (top) and relative values (bottom) of root length of different genotypes described in (C). Relative values of the root length of each genotype grown on ABA-containing medium are normalized relative to the value of the corresponding genotype at 0 μM ABA, which is taken as 100%. Values are the mean±SE of three biological determinations, and different letters represent significant differences at *P*<0.05 (Duncan’s multiple range test).

CRK4, CRK19 and CRK20 are homologous proteins of CRK5 (Fig. 8A; Supplementary Fig. S8). We observed that the *CRK4*- and *CRK19*-overexpression lines, like the *CRK5*-overexpressing lines, displayed an ABA-hypersensitive phenotype in early seedling growth (Fig. 8B–D). However, *CRK20*-overexpression lines exhibited a wild-type ABA response in early seedling growth (Fig. 8B–D). Further experiments showed that two knock-down mutants of *CRK19*, *crk19-1* and *crk19-2*, showed no ABA-related phenotype in early seedling growth (see Supplementary Fig. S9A–C). These data suggest that CRK4 and CRK19, together with CRK5, redundantly regulate ABA signaling, whereas CRK20 is not involved in ABA-mediated early seedling growth arrest.

### Overexpression of *CRK4* results in ABA hypersensitivity in stomatal movement and enhances drought tolerance

**Fig. 9.**
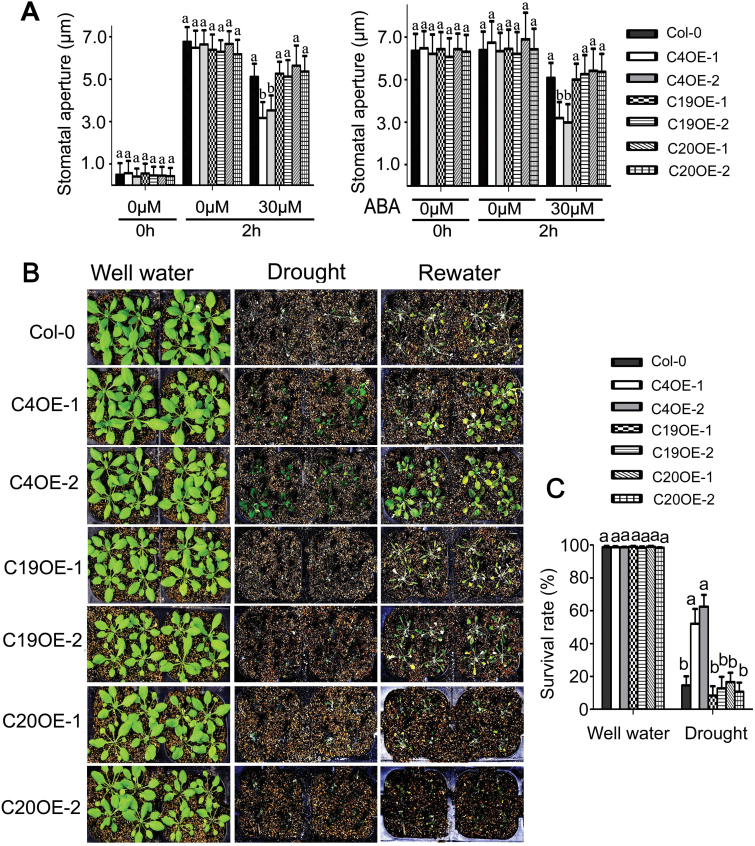
Overexpression of *CRK4*, but not *CRK19* or *CRK20*, results in ABA hypersensitivity in stomatal movement and increases plant drought tolerance. (A) ABA-induced inhibition of stomatal opening (left) and promotion of stomatal closure (right) in wild-type Col-0 plants, *CRK4* (C4OE1, C4OE2), *CRK19* (C19OE1, C19OE2) and *CRK20* (C20OE1, C20OE2) transgenic lines. The experiments were repeated five times with similar results. The values are the mean±SE from 60 stomata for each time point, and different letters represent significant differences at *P*<0.05 (Duncan’s multiple range test). (B) Test of drought tolerance of the different genotypes described above. Plants were well watered (control, ‘Well water’) or drought stressed by withholding water (‘Drought’) for 16 d and then re-watered (‘Rewater’). The experiments were repeated five times, and at least 30 plants per individual line were used for each experiment. (C) Survival rates of the plants described in (B). The values are the mean±SE of three biological determinations, and different letters represent significant differences at *P*<0.05 (Duncan’s multiple range test).

We further found that the *CRK4*-overexpression lines, like the *CRK5*-overexpressing lines, showed ABA-hypersensitive phenotype in stomatal movement (Fig. 9A) and enhanced drought tolerance (Fig. 9B, C), but neither *CRK19*- nor *CRK20*-overexpression lines showed significantly different phenotypes in stomatal movement in response to ABA and in drought response compared with the wild-type plants (Fig. 9A–C). These data suggest that CRK4, but not CRK19, cooperates with CRK5 to regulate ABA response of guard cells as well as drought response, and that the function of CRK19 is only involved in the CRK5-mediated ABA response in early seedling growth.

### WRKY18 and WRKY40, but not WRKY60, bind to the promoter of the *CRK5* gene, while all the three WRKYs inhibit promoter activity of this gene

**Fig. 10.**
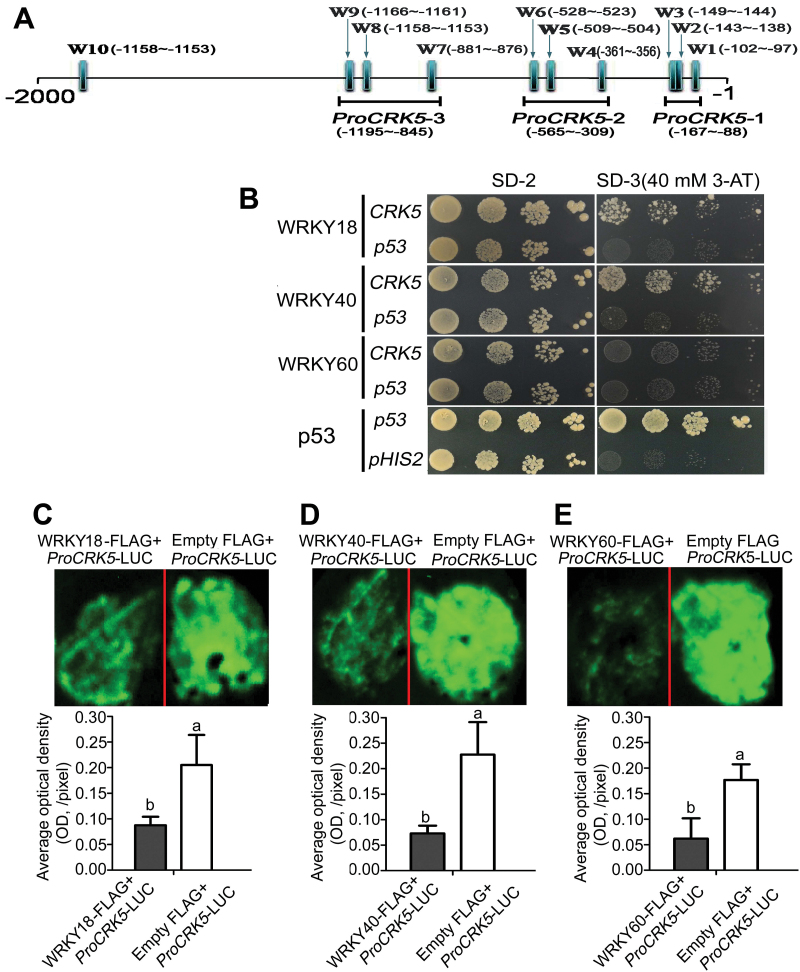
Test of the interaction of WRKY18, WRKY40 and WRKY60 with the promoter of the *CRK5* gene. (A) Promoter diagram of the *CRK5* gene. W1–W10 represent the W-box numbered from left to right with their location sites relative to the start codon (ATG). The segments marked with *ProCRK5-1*, *ProCRK5-2* and *ProCRK5-3* indicate the probe fragments used in the gel shift assays described in Fig. 11. (B) Yeast one-hybrid assays to test the interaction of WRKYs with the *CRK5* promoter. Yeast cells were co-transformed with pGADT7 prey vector containing WRKY18, WRKY40, or WRKY60 and pHIS2 bait vector containing the promoter of *CRK5*. The corresponding transformation with pGADT7 prey vector containing WRKY18, WRKY40, or WRKY60 and pHIS2 bait vector containing *p53* promoter fragment were used as negative controls. Co-transformation of pHIS2-*p53* and pGADT7-*p53* was used as positive control, and co-transformation of pGADT7-*p53* and empty pHIS2 was used as its own negative control. Three 10-fold series dilutions were dropped vertically for each assay on SD-2 medium (synthetic dropout medium lacking Leu, Trp) and SD-3 medium (synthetic dropout medium lacking Trp, Leu, His) supplemented with 40mM 3-aminotriazole (3-AT). All the experiments were repeated five times with the same results. (C) WRKY18, (D) WRKY40 and (E) WRKY60 inhibit the transcription activity of the *CRK5* promoter in tobacco system, assayed with luciferase (LUC) imaging. Tobacco leaves were co-transformed with the constructs *ProCRK5*-*LUC* plus *WRKY18-FLAG* or *ProCRK5-LUC* plus empty *FLAG* (C), or with the constructs *ProCRK5*-*LUC* plus *WRKY40-FLAG* or *ProCRK5-LUC* plus empty *FLAG* (D), or with the constructs *ProCRK5*-*LUC* plus *WRKY60-FLAG* or *ProCRK5-LUC* plus empty *FLAG* (E). Top panels in (C), (D) and (E): LUC fluorescence imaging. Bottom panels in (C), (D) and (E): optical densities calculated with the ImageJ software. The experiments were repeated three times with similar results. Each value for the columns in (C), (D) and (E) are the mean±SE of three biological determinations, and different letters represent significant differences at *P*<0.05 (Duncan’s multiple range test).

Sequence analysis shows that there are ten W-boxes in the putative promoter region of the *CRK5* gene (Fig. 10A), and we tested whether the three closely related ABA-responsive transcription factors (Shang *et al.*, 2010; Liu *et al.*, 2012, 2013; Yan *et al.*, 2013; Geilen and Böhmer, 2015), WRKY18/40/60, regulate *CRK5* expression. In the yeast one-hybrid system, yeast cells co-transformed with pGADT7 prey vector containing WRKY18 or WRKY40 and pHIS2 bait vector containing the promoter of *CRK5* grew well in SD-3 medium (lacking Trp, Leu, and His) supplemented with 40mM 3-AT (Fig. 10B), indicating a possible interaction between WRKY18/40 and the *CRK5* promoter. However, yeast cells co-transformed with pGADT7 prey vector containing WRKY60 and pHIS2 bait vector containing the promoter of *CRK5* could not grow in the SD-3 medium supplemented with 40mM 3-AT (Fig. 10B), suggesting that WRKY60 do not bind to the promoter of *CRK5*.

We assayed possible effects of the three WRKYs on the promoter activities of the *CRK5* gene by co-transforming tobacco leaves with the *CRK5* promoter linked to a *LUC* reporter gene together with *WRKY18*, *WRKY40*, or *WRKY60* gene. We observed that all the three WRKYs showed inhibitory effects on the activity of the *CRK5* promoter, as shown by LUC fluorescence intensity (Fig. 10C–E).

**Fig. 11.**
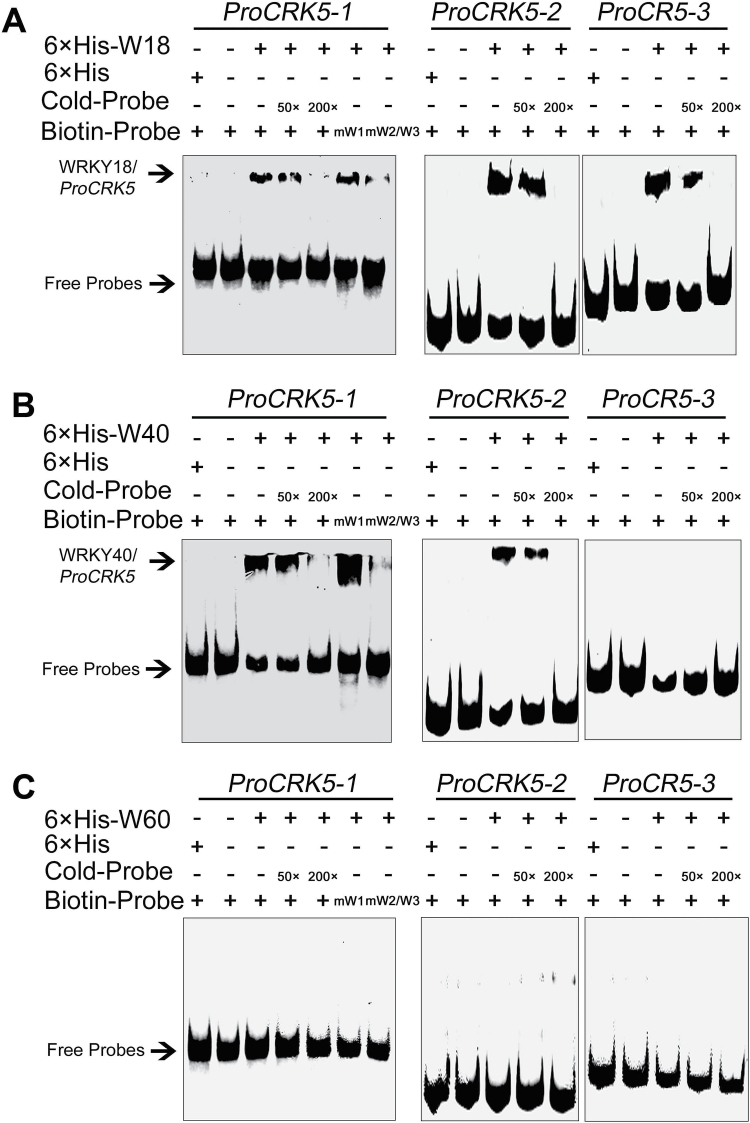
Gel shift assays to test interaction of WRKY18, WRKY40 or WRKY60 with the promoter of *CRK5* gene. (A) WRKY18 binds to the *ProCRK5-1*, *ProCRK5-2* and *ProCRK5-3* fragments. 6×His-W18 indicates the purified 6×His-WRKY18 fusion protein; 6×His, the 6×His tag peptide served as negative control; Biotin-Probe, the biotin labeled *CRK5* promoter fragments *ProCRK5-1*, *ProCRK5-2*, *ProCRK5-3*; mW1 and mW2/W3, the two mutant forms in W-boxes (W1 single mutation and W2/W3 double mutation) of biotin labeled *ProCRK5-1* with W-box mutation at W1: TTGACT→TTAAAT, and W-box mutation at W2/W3: TTGACCTTGACT→TTAAACTTAAAT; Cold-Probe, the three unlabeled *CRK5* promoter fragments; 50× and 200×, 50-fold or 200-fold cold-probe relative to the labeled probes added, respectively, for binding competition; Free-Probes, the labeled probes that do not bind the WRKY protein; WRKY18/*ProCRK5*, the shift bands of the complex of WRKY18 protein with corresponding *ProCRK5* fragments. Each experiment was repeated three times with the same results. (B) WRKY40 binds to the *ProCRK5-1*, *ProCRK5-2* fragments, but does not bind to the *ProCRK5-3* sequence. 6×His-W40 denotes the purified 6×His-WRKY40 fusion protein; WRKY40/*ProCRK5*, the shift bands of the complex of WRKY40 protein with the corresponding *ProCRK5* fragments. Other symbols are the same as described above in (A). Each experiment was repeated three times with the same results. (C) WRKY60 does not bind to any of the three promoter segments of *CRK5* gene. 6×His-W60 denotes the purified 6×His-WRKY60 fusion protein, and other symbols are the same as described above in (A). Each experiment was repeated three times with the same results.

We performed the gel shift assays (GSA) in which three domains of the *CRK5* promoter were used (*ProCRK5-1*, *ProCRK5-2*, *ProCRK5-3*) (Fig. 10A). 6×His tagged recombinant WRKY proteins were expressed and purified in *E. coli* (see Supplementary Fig. S10A–C). We showed that WRKY18 bound to all the three domains and WRKY40 bound to the *ProCRK5-1* and *ProCRK5-2* domains, while the binding was reduced by adding increasing amounts of unlabeled competitors (Fig. 11A, B). When a mutation of W-box 1 was introduced into the *ProCRK5-1* domain, WRKY18 and WRKY40 still bound to the mutant form of the fragment (Fig. 11A, B). However, when double mutation of W-box 2 and W-box 3 was introduced into the *ProCRK5-1* domain, WRKY18 and WRKY40 could scarcely bind to this domain (Fig. 11A, B), suggesting that W-box 2 and W-box 3 are two core *cis*-regulatory elements to which WRKY 18 and WRKY40 bind. WRKY60 showed no binding affinity to any of the three fragments (Fig. 11C). As negative controls, we purified empty 6×His protein (Supplementary Fig. S10D) and observed no shift bands in the control assays with 6×His protein (Fig. 11A–C). Together, these data indicate that WRKY18 and WRKY40, but not WRKY60, bind to the *CRK5* promoter, which is consistent with data from the yeast-one hybrid, but not completely consistent with the data from assays in tobacco leaves where WRKY60, like WRKY18 and WRKY40, showed an inhibitory effect on the activity of *CRK5* promoter. This may be due to a complex cooperation among these three homologous WRKYs, which interact functionally in a manner of redundancy, antagonistic, and distinct roles (Xu *et al.*, 2006; Liu *et al.*, 2012; Yan *et al.*, 2013).

### Triple loss-of-function mutation of *WRKY18/40/60* enhances expression level of the *CRK5* gene

**Fig. 12.**
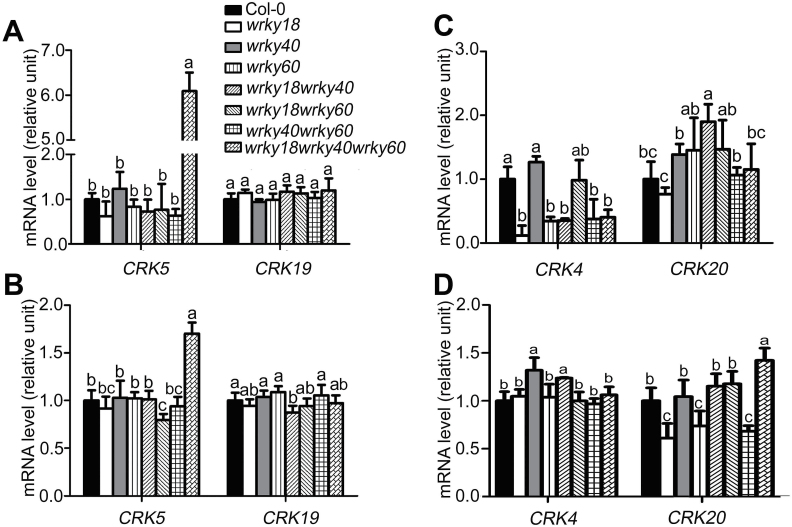
Expression of *CRK4*, *CRK5*, *CRK19* and *CRK20* genes in *wrky* loss-of-function mutants. (A–D) Two-week-old seedlings grown on MS medium (A, C) or rosette leaves of 4-week-old seedlings (B, D) were sampled for RNA extraction. The transcription levels of *CRK4*, *CRK5*, *CRK19* and *CRK20* were assayed in the *wrky* single, double (*wrky40 wrky18*, *wrky18 wrky60*, and *wrky40 wrky60*), and triple (*wrky40 wrky18 wrky60*) mutants by real-time PCR. *Actin2/8* was used as internal control. Each value is the mean±SE of three independent experiments, and the letters indicate significant differences at *P*<0.05 (Duncan’s multiple range test).

We tested the mRNA level of *CRK5* in *wrky* single, double (*wrky40 wrky18*, *wrky18 wrky60*, and *wrky40 wrky60*), and triple (*wrky40 wrky18 wrky60*) mutants, and observed that neither *WRKY* single nor double mutations affected the *CRK5* transcription level, which, however, was significantly enhanced in the *wrky40 wrky18 wrky60* triple mutant (Fig. 12A, B). This suggests that the three WRKYs act cooperatively to inhibit *CRK5* expression. We also assayed the expression levels of three homologous genes of *CRK5*, *CRK4*, *CRK19* and *CRK20*, and found that expression of *CRK4* was down-regulated in several *wrky* mutants in 2-week-old young seedlings, contrarily to *CRK5* (Fig. 12C), suggesting a positive regulation, but this effect was lost in the mature plants (4 weeks old) (Fig. 12D). These differences between *CRK4* and *CRK5* genes in the transcriptional regulation by the WRKYs suggest that a complex mechanism may be involved in the processes to maintain homeostasis of the CRK protein amounts to balance ABA signaling. Globally, however, there was no marked alteration of the expression levels of the three homologous *CRK* genes with loss-of-function of these WRKYs even in the *wrky40 wrky18 wrky60* triple mutant (Fig. 12A–D).

Given that WRKY18/40/60 are supposed to be regulated by ABA (Shang *et al.*, 2010; Geilen and Böhmer, 2015), we tested whether expression of *CRK5* is induced by ABA, and found that the expression levels of *CRK5* were not significantly affected by ABA treatment (see Supplementary Fig. S11). This suggests that a complex feed-back and feed-forward mechanism may function to regulate homeostasis of *CRK5* expression in response to ABA.

## Discussion

### CRK5 is a potentially positive regulator of ABA signaling

In this study, we observed that the *CRK5*-overexpression lines displayed ABA-hypersensitive phenotypes in ABA-induced post-germination growth arrest, promotion of stomatal closure and inhibition of stomatal opening (Figs 1–3; Supplementary Figs. S2 and S3). Consistent with the hypersensitivity of guard cells in response to ABA, the *CRK5*-overexpression lines showed drought tolerance (Fig. 3). Given that plant drought tolerance has been shown to be generally linked to reduction of growth and productivity, we observed, interestingly, that these *CRK5*-overexpression lines showed normal growth and productivity during mature stages under non-stressful conditions, thought the early seedlings showed shorter roots (Figs 1–4; Supplementary Figs S2 and S3), which suggests that CRK5 is likely to be useful in agriculture to improve crop tolerance to drought by transgenic manipulation. The enhancement of drought tolerance of the *CRK5*-overexpression lines may be attributed partly to an ABA-hypersensitive response of stomatal movement, which can minimize water transpiration from leaves under drought conditions that induce ABA accumulation (Leung and Giraudat 1998; Zhu, 2002; Kwak *et al.*, 2008). However, ABA regulates plant adaptation to drought by regulating both water balance and osmotic stress/cellular dehydration tolerance, which is associated with both guard cell regulation and the induction of dehydration tolerance genes in nearly all cells (Zhu, 2002). Therefore, up-regulation of a set of ABA- and drought-responsive genes in the *CRK5*-overexpression lines (Fig. 7), which potentially induces cellular dehydration tolerance, additionally explains the mechanism of enhanced drought tolerance resulting from *CRK5* overexpression.

Transgenic lines overexpressing the mutant form of *CRK5* ^*K372E*^ showed wild-type ABA responses and drought sensitivity (Figs 1–3; Supplementary Figs S2 and S3). These observations reveal that CRK5 mediates ABA signaling through catalyzing phosphorylation of downstream targets by the cytoplasmic kinase domain in which the 372nd amino acid plays an essential role, and on the other hand, these data provide substantial, supporting evidence for involvement of CRK5 in ABA signaling.

Additionally, transgenic lines overexpressing the *CRK5* homologous genes, *CRK4* and *CRK19*, also exhibited ABA-hypersensitive phenotypes, whereas overexpression lines of *CRK20* showed wild-type ABA responses in ABA-induced inhibition of early seedling growth, suggesting that CRK4 and CRK19, but not CRK20, function redundantly together with CRK5 in an overlapping manner in ABA signaling (Fig. 8). Interestingly, overexpression of *CRK4* also results in ABA hypersensitivity in stomatal movement and enhances drought tolerance (Fig. 9), suggesting that CRK5 works, together with CRK4, to regulate guard cell response to ABA and to drought. We failed to obtain double or triple mutants of these *CRK* genes because *CRK5* and *CRK19* localize to the same chromosome with a relative close genetic distance and there is currently no *crk4* or *crk20* loss of function mutant in the public biological resources. Nevertheless, the possible functional redundancy of CRK4, CRK5 and CRK19 explains, at least partly, the wild-type ABA responses in the *crk5* loss-of-function mutants and *crk19* knockdown mutants (Fig. 1; Supplementary Fig. S9). Taken all together, in the present experiment, we identified a cysteine-rich receptor-like protein kinase, CRK5, as a potentially positive regulator of ABA signaling in early seedling growth and stomatal movement.

### How may CRK5 function in ABA signaling?

Previous studies have reported that the three closely related WRKYs function as negative regulators of ABA signaling by repressing expression of a subset of ABA-responsive genes such as *ABI4* and *ABI5*, in which WRKY60 plays a role in balancing the binding activities of WRKY18 and WRKY40 to the *ABI4* and *ABI5* promoters (Shang *et al.*, 2010; Liu *et al.*, 2012). Studies in plant response to pathogens showed that WRKY60 may function through interaction with WRKY18 and WRKY40 to promote or decrease their binding affinities to the promoters of their target genes (Xu *et al.*, 2006; Shen *et al.*, 2007; Wenke *et al.*, 2012). Consistently, in this study, we showed that WRKY18, WRKY40 and WRKY60 transcription factors cooperatively repress *CRK5* gene expression (Figs 10–12). WRKY18 and WRKY40, but not WRKY60, directly bind to the *CRK5* promoter (Figs 10 and 11), but all the three WRKYs can repress promoter activity of *CRK5* in a system of tobacco leaves (Fig. 10), suggesting that WRKY60 may interact, directly or indirectly, with other transcription factor(s) to act on the CRK5 promoter. The *CRK5* gene is markedly relieved from inhibition only in the *wrky18 wrky40 wrky60* triple mutant, but not in *wrky18*, *wrky40* or *wrky60* single or any double mutants (Fig. 12), indicating that a complex cooperation mechanism of the three WRKYs occurs in the repression of *CRK5* expression, which is likely to function upstream of the CRK5-mediated ABA signaling.

CRK5 is a typical RLK member with an extracellular domain, a transmembrane domain, and a cytoplasmic protein kinase domain (see Supplementary Figs S7 and S8). In the present study, we showed that the cytoplasmic kinase domain may be of importance for ABA signaling (Figs 1–3; Supplementary Figs S2 and S3). Therefore, identification of the substrates of CRK5 is important for understanding of the CRK5-mediated ABA signaling. The present experiment provided genetic evidence that CRK5 may function upstream of ABI2 in ABA signaling (Fig. 5), but whether ABI2 is a direct downstream regulator of CRK5 remains unknown. It has been known that the protein phosphatase ABI2, functioning downstream of the cytosolic ABA receptors PYR/PYL/RCARs, is one of the central players in this PYR/PYL/RCAR-mediated, core ABA signaling pathway (Fujii *et al.*, 2009; Ma *et al.*, 2009; Park *et al.*, 2009). So, further studies are needed to determine whether and how CRK5 is involved in the PYR/PYL/RCAR-mediated ABA signaling pathway and to understand the complex ABA signaling network.

## Supplementary data

Supplementary data are available at *JXB* online

Figure S1. Alignment of the conserved cytoplasmic kinase domain of the *Arabidopsis* receptor-like protein kinases CRK5, CRK36, ARCK1, BAK1 and RPK1.

Figure S2. Overexpression of *CRK5*, but not its mutated form *CRK5* ^*K372E*^, results in ABA hypersensitive phenotype in early seedling growth.

Figure S3. ABA-induced inhibition of seedling growth is negatively correlated with *CRK5* expression levels.

Figure S4. Transgenic line expressing *GFP* tag alone shows wild-type ABA response in early seedling growth.

Figure S5. The precise T-DNA insertion site of the *CRK5* and *ABI2* transgenic lines.

Figure S6. Overexpression of *CRK5* in *aba2* mutant background partially restored drought tolerance of *aba2* mutant.

Figure S7. Prediction of the potential transmembrane domains in CRK5 protein.

Figure S8. Alignment of the amino acids of the *Arabidopsis* CRK4, CRK5, CRK19 and CRK20.

Figure S9. Two knock-down mutants of *CRK19*, *crk19-1* and *crk19-2*, showed no ABA-related phenotype in early seedling growth.

Figure S10. Identification of the recombined proteins used in this study.

Figure S11. Test of the effects of ABA treatment on *CRK5* gene expression.

Table S1. PCR primers used in this study.

Supplementary Data
