# Supplementary material for: Overexpression of an Arabidopsis cysteine-rich receptor-like protein kinase, CRK5, enhances abscisic acid sensitivity and confers drought tolerance
Source: J Exp Bot. 2016 Jul 12;67(17):5009–27. doi: 10.1093/jxb/erw266 (PMC5014153; doi:10.1093/jxb/erw266)
Supplement: Supplementary Data [file supp_67_17_5009__index.html]

Overexpression of an Arabidopsis cysteine-rich receptor-like protein kinase, CRK5, enhances abscisic acid sensitivity and confers drought tolerance — Overexpression of an Arabidopsis cysteine-rich receptor-like protein kinase, CRK5, enhances abscisic acid sensitivity and confers drought tolerance — Supplementary Data 

# Overexpression of an Arabidopsis cysteine-rich receptor-like protein kinase, CRK5, enhances abscisic acid sensitivity and confers drought tolerance

## Supplementary Data

Data files

- supplementary\_figures\_S1\_S11\_table\_S1.pdf - Supplementary Data
