# Supplementary material for: Overexpression of an Arabidopsis cysteine-rich receptor-like protein kinase, CRK5, enhances abscisic acid sensitivity and confers drought tolerance
Source: J Exp Bot. 2016 Jul 12;67(17):5009–27. doi: 10.1093/jxb/erw266 (PMC5014153; doi:10.1093/jxb/erw266)
Supplement: Supplementary Data [file supp_erw266_supplementary_figures_S1_S11_table_S1.pdf]

Supplementary Data

Lu *et al.*, Overexpression of an *Arabidopsis* cysteine-rich receptor-like protein kinase, CRK5, enhances abscisic acid sensitivity and confers drought tolerance

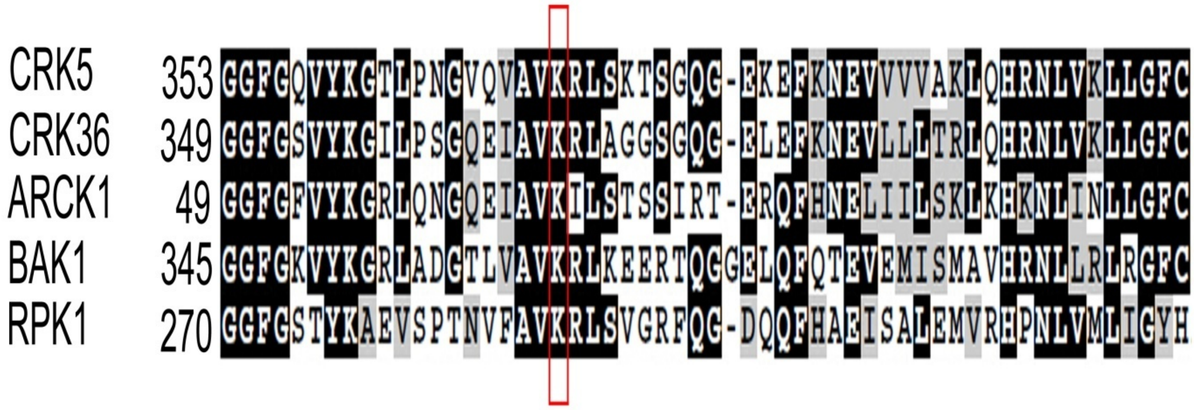

Supplementary Fig. S1. Alignment of the conserved cytoplasmic kinase domain of the *Arabidopsis* receptor-like protein kinases CRK5, CRK36, ARCK1, BAK1 and RPK1. The lysine labeled with red box is the conserved lysine in the cytoplasmic kinase domain of these CRKs, and the 372th amino acid is the conserved lysine in the kinase domain of CRK5 protein. Black boxes correspond to amino acid residues strictly conserved in the five sequences, and gray boxes correspond to similar amino acid residues. Gaps are labeled with dash lines to maximize the alignment.

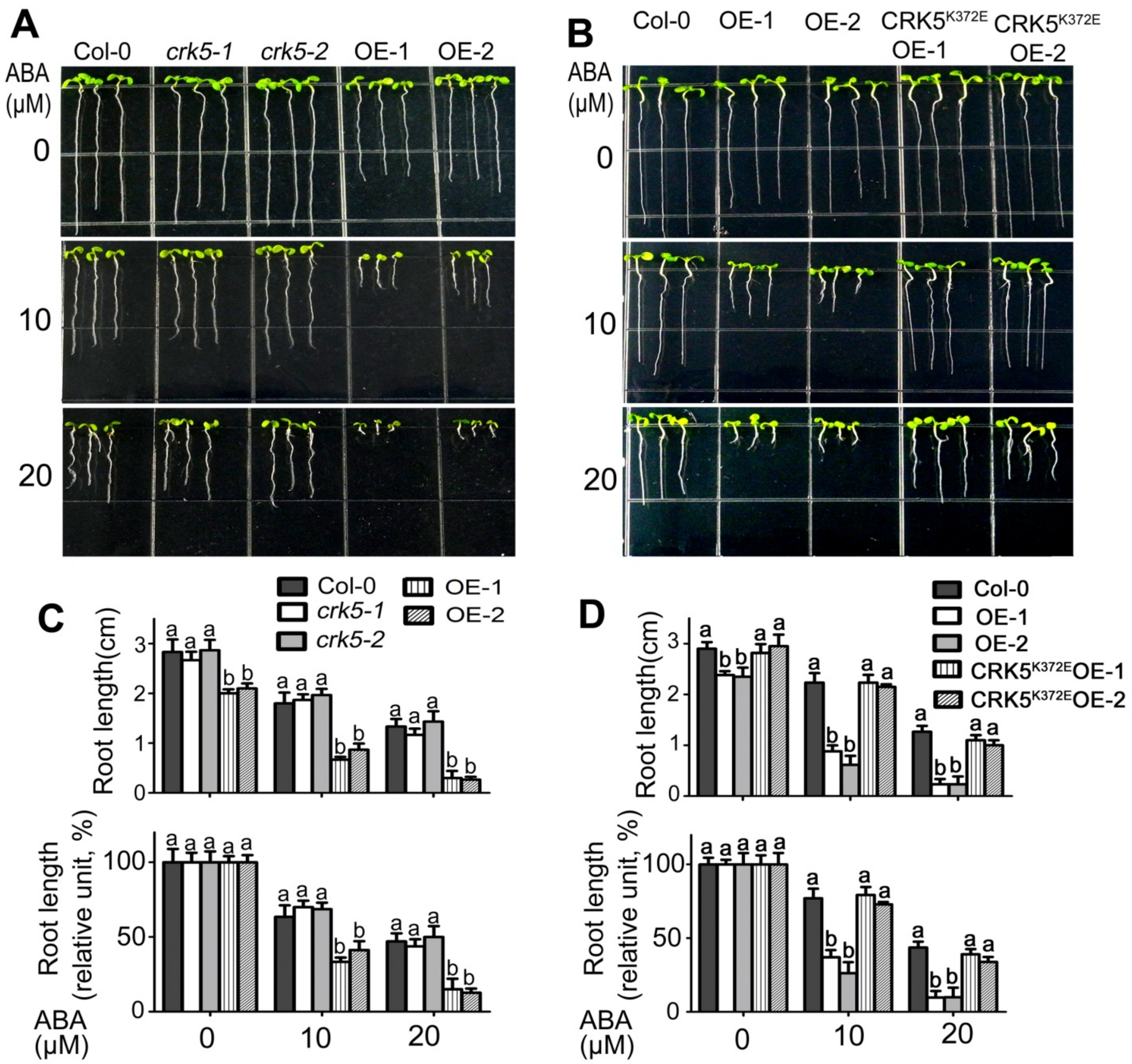

Supplementary Fig. S2. Overexpression of *CRK5*, but not its mutated form *CRK5^{K372E}*, results in ABA hypersensitive phenotype in early seedling growth. **(A)** and **(B)** Root growth of wild-type Col-0, *crk5-1*, *crk5-2*, *CRK5*-transgenic lines OE-1 and OE-2 (A), or Col-0, OE-1, OE-2, and *CRK5^{K372E}*-transgenic lines CRK5^{K372E}OE-1 and CRK5^{K372E}OE-2 (B) growing on ABA-free (0 μM) or (±)ABA-containing (10 and 20 μM) MS medium. Seeds were planted in ABA-free medium, subjected to a 3-d-stratification, and 60-h-old-germinating seeds/early seedlings were transferred to ABA-free (0 μM) or (±)ABA-containing (10 and 20 μM) MS medium and continued to grow 10 d before investigation. The experiments were repeated three times with similar results. **(C)** and **(D)** Statistical analysis of absolute (top) and relative values (bottom) of root length of different genotypes described in (A) and (B), respectively. Relative values of the root length of each genotype grown on MS medium containing 10 and 20 μM (±)ABA are normalized relative to the value of the corresponding genotype at 0 μM (±)ABA, which is taken as 100%. Values are the mean ± SE of three biological determinations, and different letters represent significant differences at $P<0.05$ (Duncan's multiple range test).

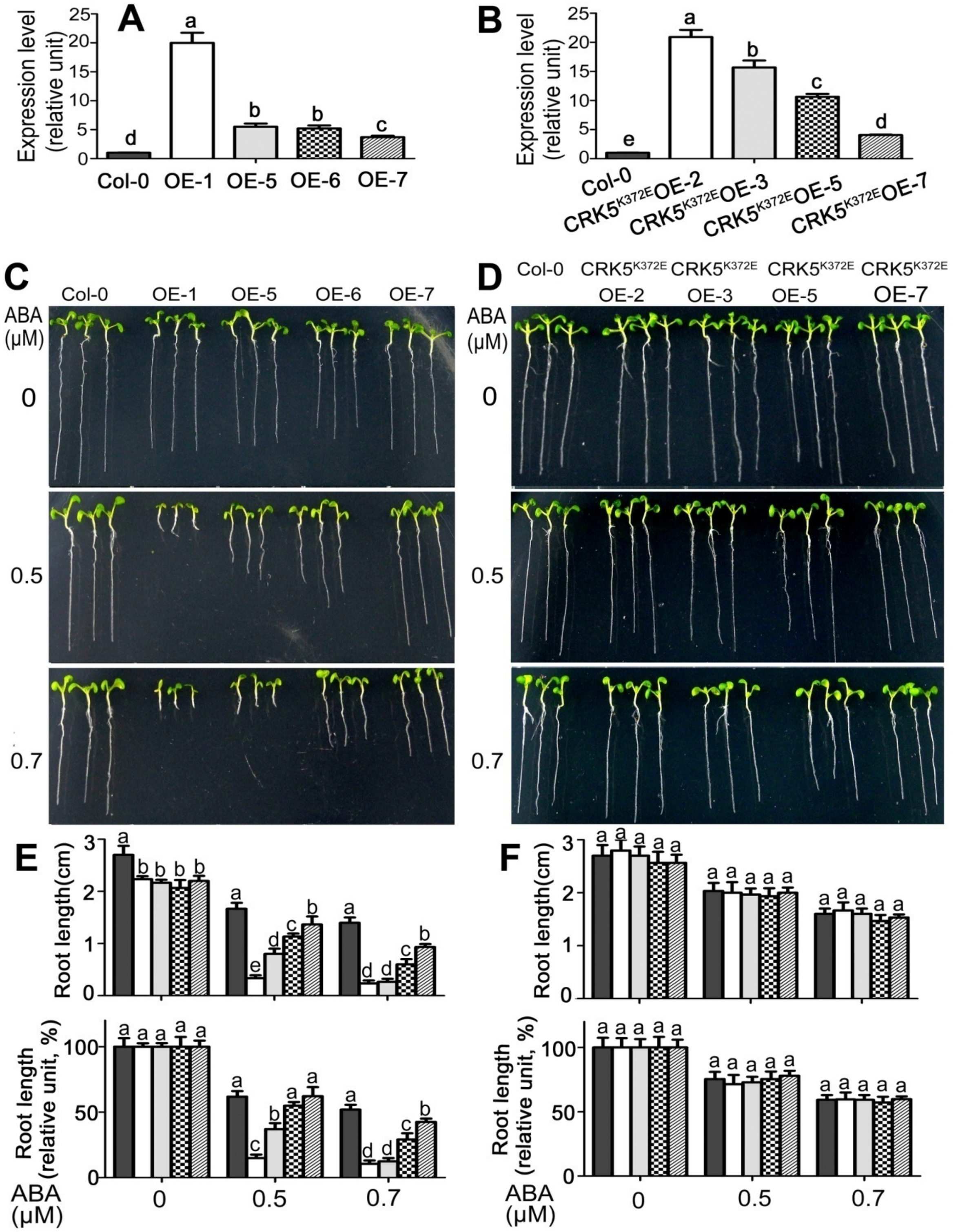

Supplementary Fig. S3. ABA-induced inhibition of seedling growth is negatively correlated with *CRK5* expression levels. **(A)** and **(B)** Real-time PCR analysis of the transgenic lines overexpressing *CRK5* (OE-1, OE-5, OE-6 and OE-7; A) or *CRK5*^{K372E} (CRK5^{K372E} OE-2, CRK5^{K372E} OE-3, CRK5^{K372E} OE-5 and CRK5^{K372E} OE-7; B). Expression level of *CRK5* or *CRK5*^{K372E} was normalized to that of *Actin2/8*, and the expression level of *CRK5* in

Col-0 was set to 1. Values are the mean ± SE of three independent biological determinations, and different letters represent significant differences at $P<0.05$ (Duncan's multiple range test). **(C)** and **(D)** Root growth of Col-0, OE-1, OE-5, OE-6, OE-7(C) or Col-0, $CRK5^{K372E}$OE-2, $CRK5^{K372E}$OE-3, $CRK5^{K372E}$OE-5 and $CRK5^{K372E}$OE-7 (D) growing on ABA-free (0 μM) or (±)ABA-containing (0.5 and 0.7 μM) MS medium. Seeds were directly planted in the medium for a 72-h stratification and germinating seeds/young seedlings continued to grow 10 d before investigation. The experiments were repeated three times with similar results. **(E)** and **(F)** Statistical analysis of absolute (top) and relative values (bottom) of root length of different genotypes described in (C) and (D), respectively. Relative values of the root length of each genotype grown on MS medium containing 0.5 and 0.7 μM (±)ABA are normalized relative to the value of the corresponding genotype at 0 μM (±)ABA, which is taken as 100%. Values are the mean ± SE of three biological determinations, and different letters represent significant differences at $P<0.05$ (Duncan's multiple range test).

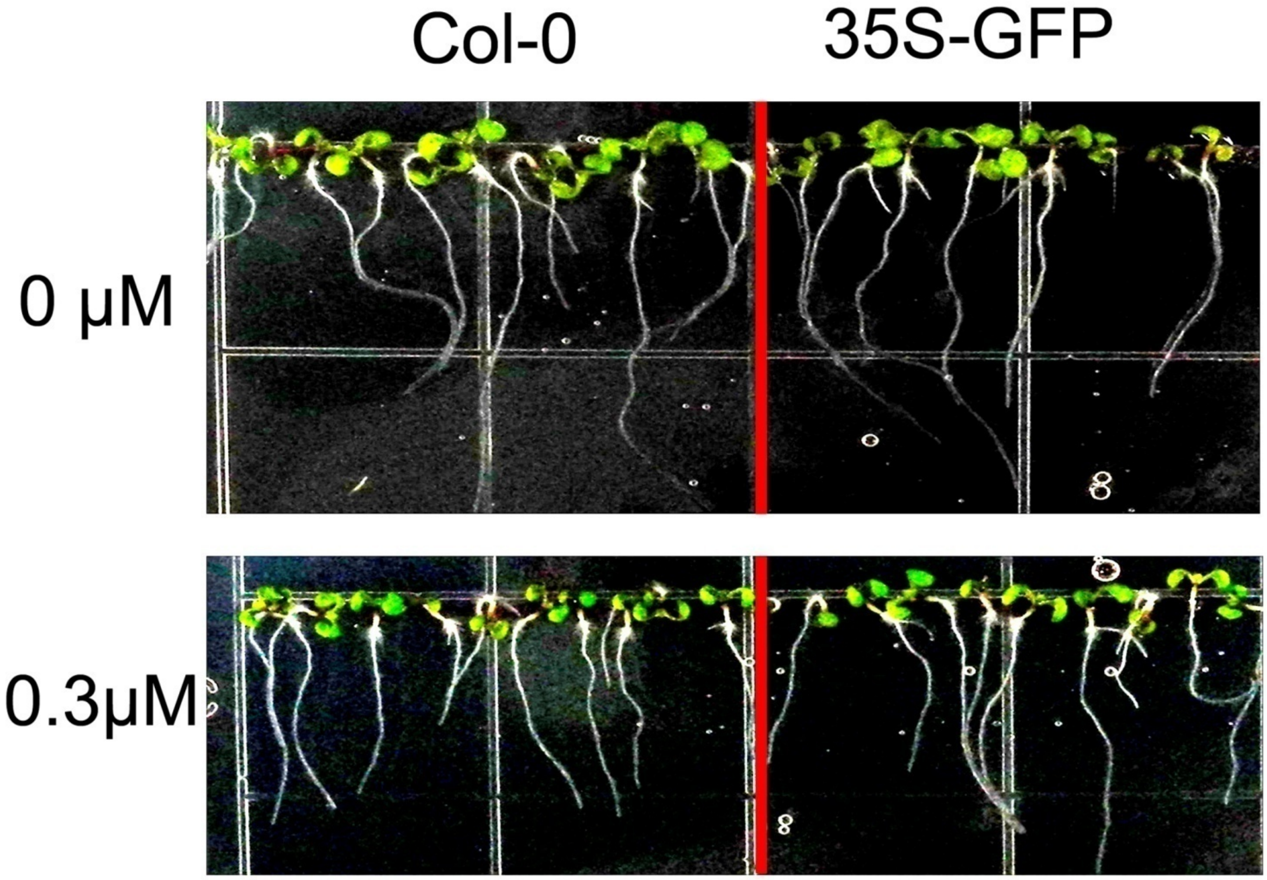

Supplementary Fig. S4. Transgenic line expressing *GFP* tag alone shows wild-type ABA response in early seedling growth. Seeds of wild-type Col-0 and *GFP*-transgenic line (35S-GFP) were directly planted on the ABA-free (0 μM) or 0.3 μM-ABA-containing MS medium, and the growth status was recorded 10 d after stratification. The experiments were repeated three times with similar results.

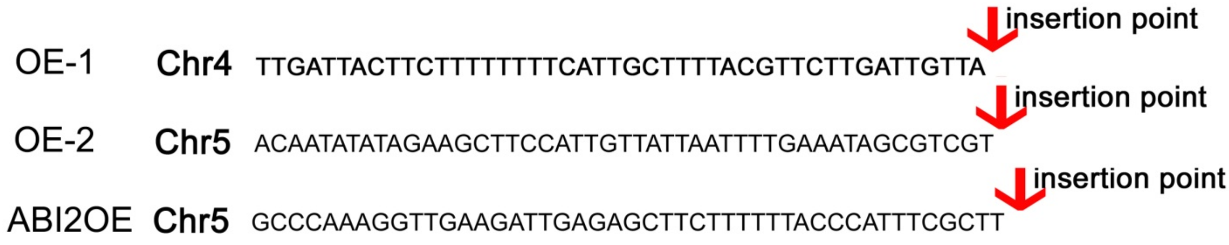

Supplementary Fig. S5. The precise T-DNA insertion site of the *CRK5* transgenic lines OE-1(top) in chromosome 4, and OE-2 (middle) in chromosome 5, and that of the *ABI2* transgenic line ABI2OE (bottom) in chromosome 5.

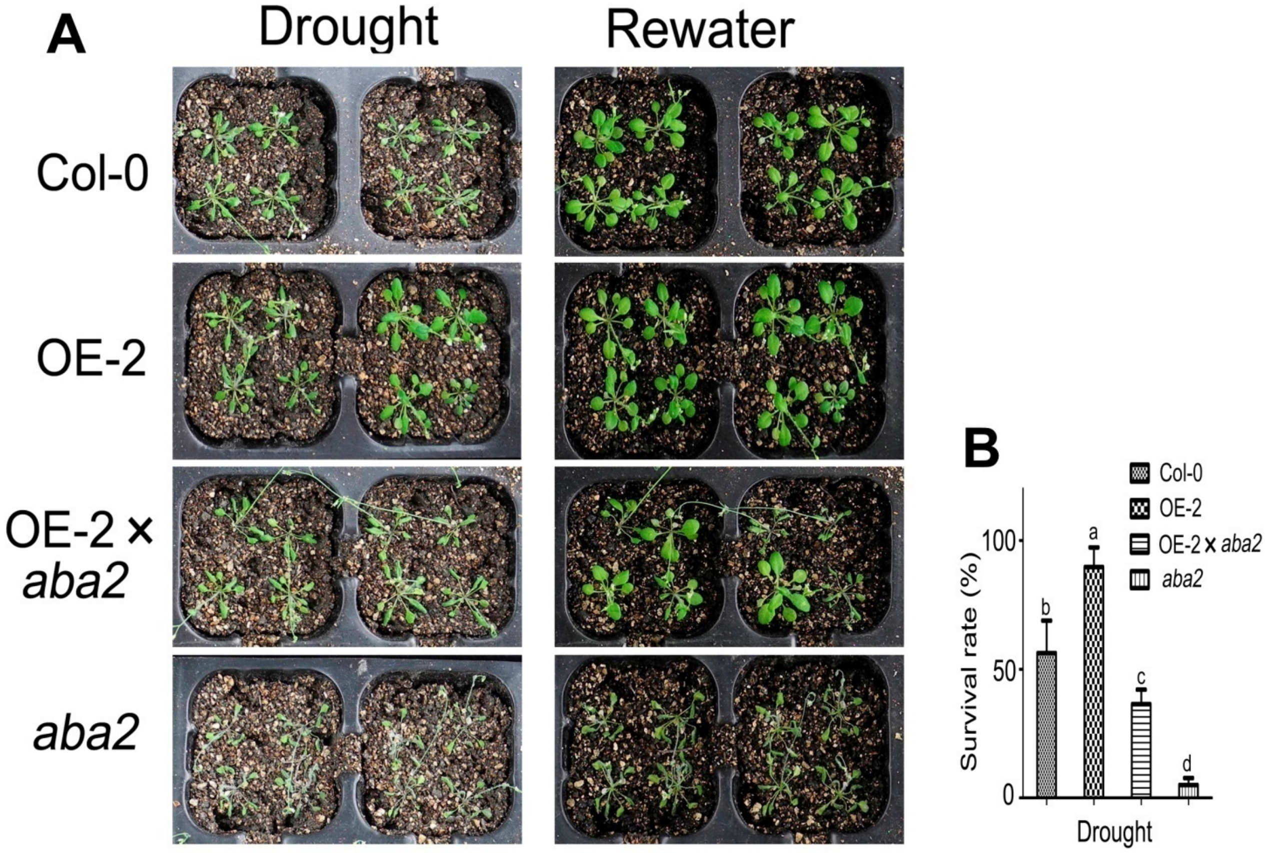

Supplementary Fig. S6. Overexpression of *CRK5* in *aba2* mutant background partially restored drought tolerance of *aba2* mutant. **(A)** Test of drought tolerance of wild type Col-0, *CRK5*-overexpression line OE-2, *aba2* mutant, and *CRK5*-overexpression line OE-2 in the *aba2* mutant background (OE-2×*aba2*). Plants were drought-stressed by withholding water ('Drought') for two weeks and then re-watered ('Rewater'). The experiments were repeated three times, and at least 30 plants per each individual line were used for each experiment. **(B)** Survival rates of the plants described in (A). The values are the mean ± SE of three biological determinations, and different letters represent significant differences at $P<0.05$ (Duncan's multiple range test).

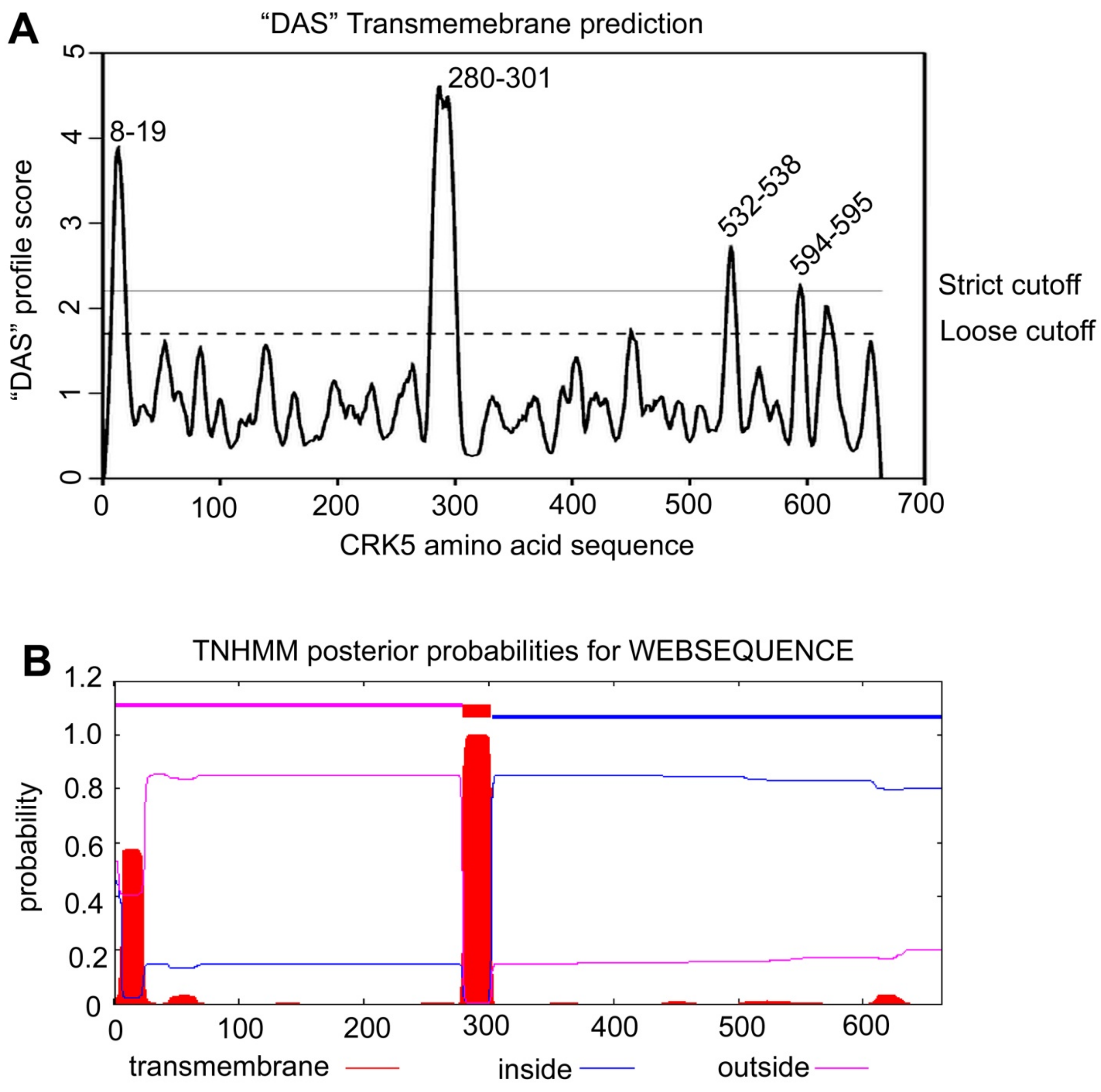

Supplementary Fig. S7. Prediction of the potential transmembrane domains in CRK5 protein. (**A**) The prediction conducted with "DAS" Transmembrane Prediction server at the web site http://www.sbc.su.se/~miklos/DAS/. There are four potential transmembrane sites with higher probability at the amino acid residues 8 to 19, 280 to 301, 532 to 538 and 594 to 595. (**B**) The prediction conducted with TMHMM algorithm at the web site http://www.cbs.dtu.dk/services/TMHMM/. The red line denotes transmembrane domain. The green line denotes inside cell domain. The pink line denotes outside cell domain.

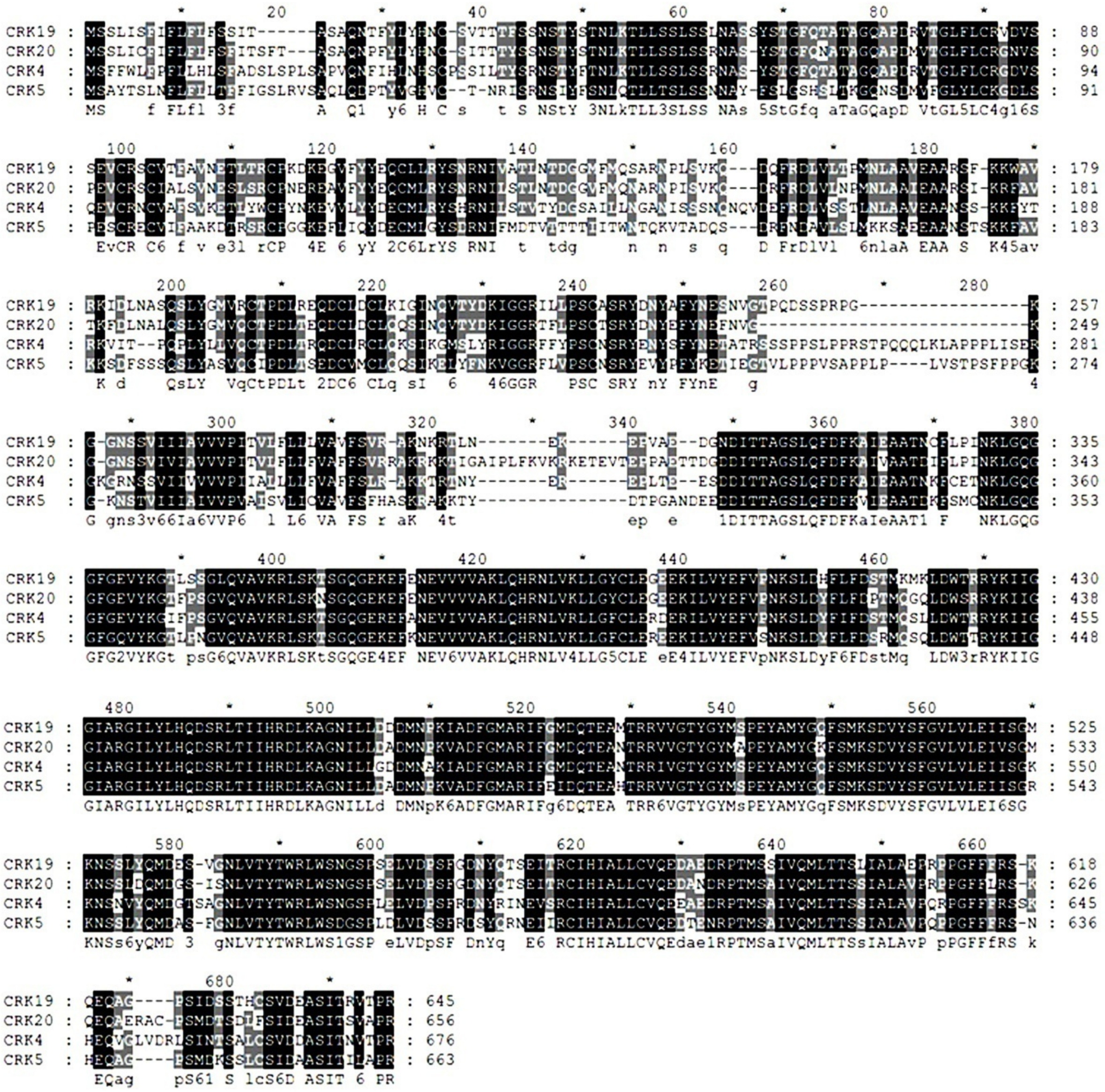

Supplementary Fig. S8. Alignment of the amino acids of the *Arabidopsis* CRK4, CRK5, CRK19 and CRK20. Sequence alignment was conducted with ClustalW software. Black boxes corresponds to amino acid residues strictly conserved in the four sequences, and gray boxes corresponds to amino acid residues strictly conserved in three of the four sequences. Gaps are labeled with dash lines to maximize the alignment. Numbers listed left of each line represent the number of amino acid residues that have been aligned in CRK4, CRK5, CRK19 and CRK20. Numbers listed up of the sequence represent the number of amino acid residues plus the number of gaps.

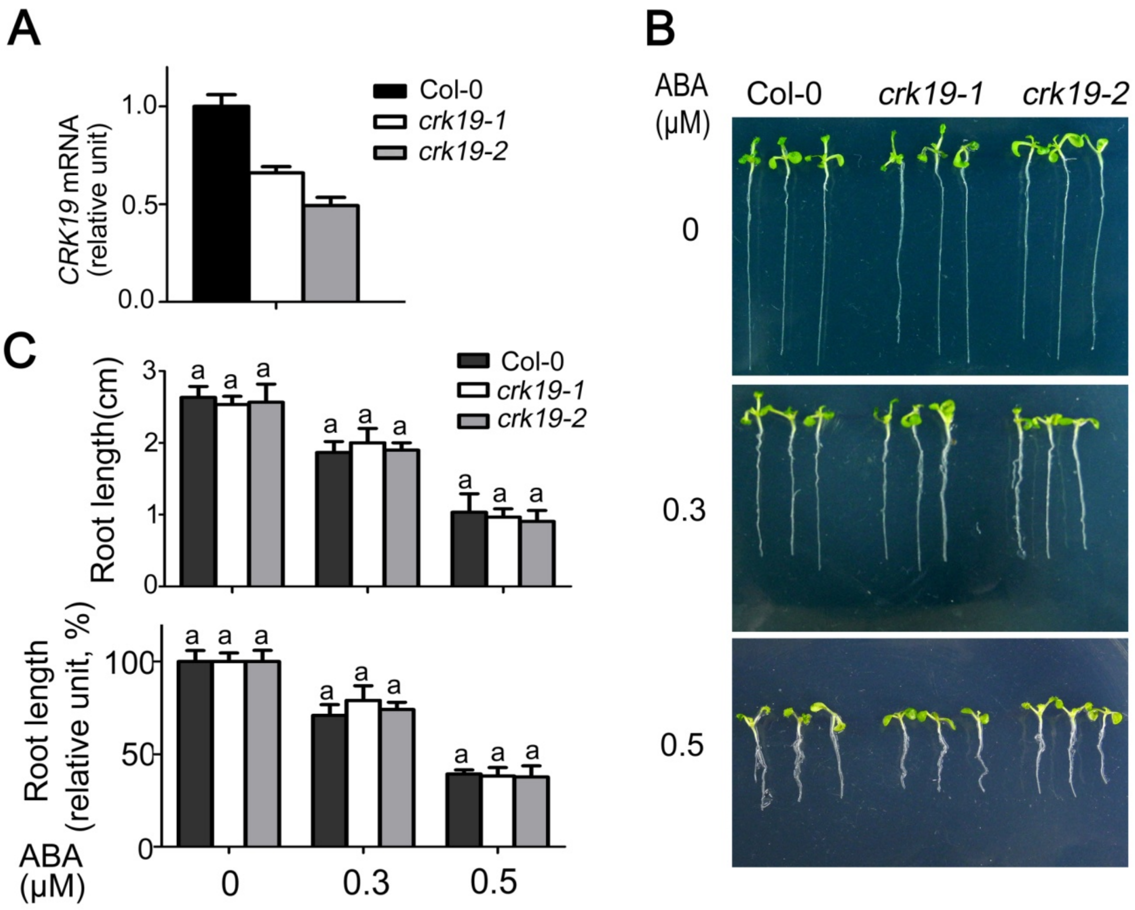

Supplementary Fig. S9. Two knock-down mutants of *CRK19*, *crk19-1* and *crk19-2*, showed no ABA-related phenotype in early seedling growth. **(A)** Real-time PCR analysis of the *CRK19* expression level in wild-type Col-0, *crk19-1* and *crk19-2* T-DNA insertion mutant plants. **(B)** Root growth of wild-type Col-0, *crk19-1* and *crk19-2* growing on ABA-free (0 µM) or (±)ABA-containing (0.3 and 0.5 µM) MS medium. Seeds were directly planted in the medium for a 72-h stratification and germinating seeds/young seedlings continued to grow 10 d before investigation. The experiments were repeated three times with similar results. **(C)** Statistical analysis of absolute (top) and relative values (bottom) of root length of different genotypes described in (B). Relative values of the root length of each genotype grown on MS medium containing 0.3 and 0.5 µM (±)ABA are normalized relative to the value of the corresponding genotype at 0 µM (±)ABA, which is taken as 100%. Values are the mean ± SE of three biological determinations, and different letters represent significant differences at $P<0.05$ (Duncan's multiple range test).

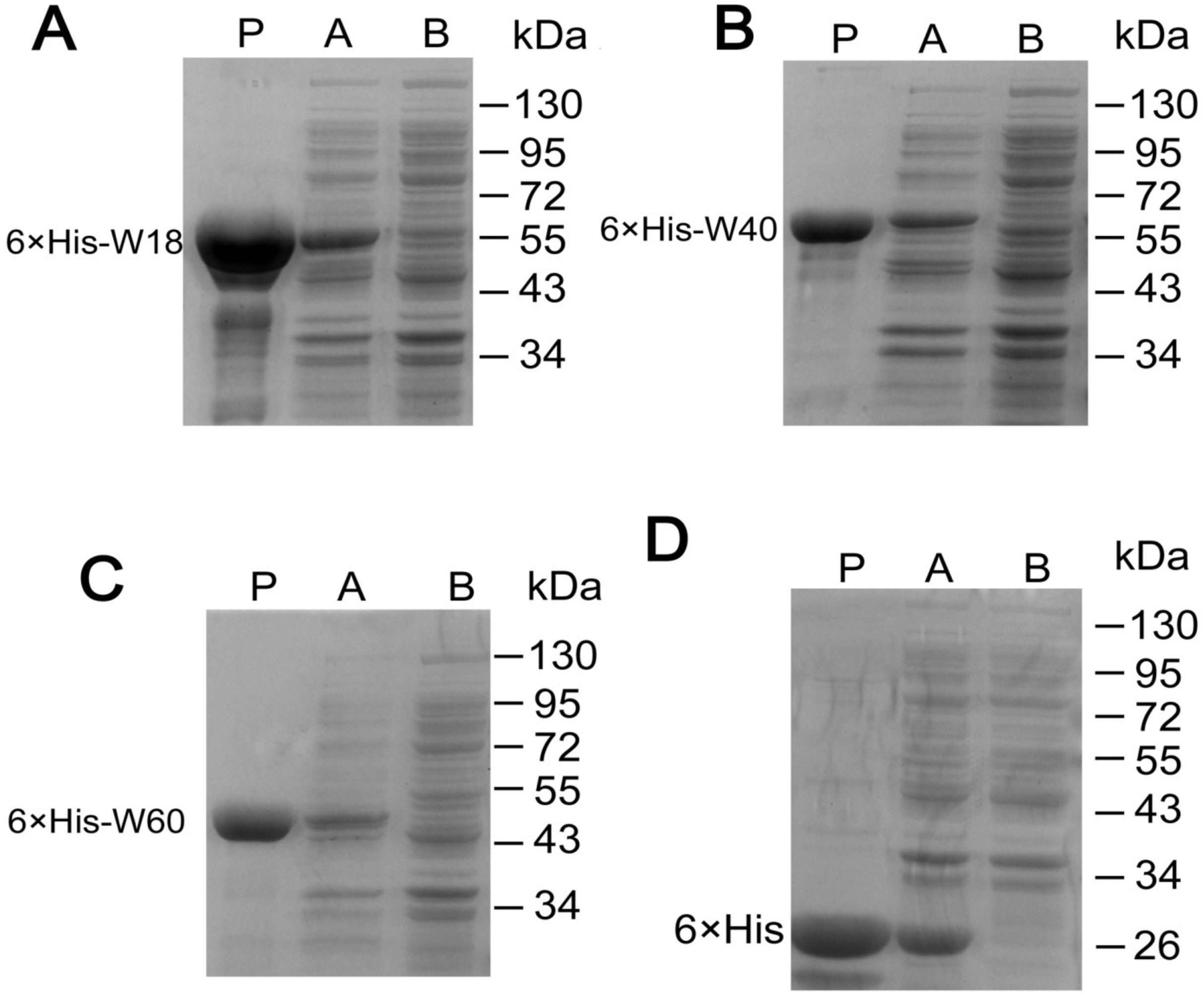

Supplementary Fig. S10. Identification of the recombined proteins used in this study. **(A)-(D)** Induction and purification of the recombinant 6×His-WRKY18 (figure A), 6×His-WRKY40 (figure B), 6×His-WRKY60 (figure C) fusion proteins or 6×His tag (figure D) in *E.coli*. kDa, kilodalton; B, total proteins before induction; A, total proteins after induction; P, total proteins after purification. The recombinant 6×His-WRKY proteins contain a Trx tag in their N-terminus, a 6×His tag and a HRV 3C protease recognition site between His tag and WRKY proteins. The control protein 6×His contains a Trx tag in its N-terminus, a S tag in its C-terminus, a 6×His tag, a HRV 3C and thrombin protease recognition sites. The predicted molecular weight of 6×His-WRKY18, 6×His-WRKY40, 6×His-WRKY60 and 6×His control protein are 52 kDa, 51 kDa, 47 kDa and 23 kDa, respectively, which are similar with their actual molecular weight as we tested.

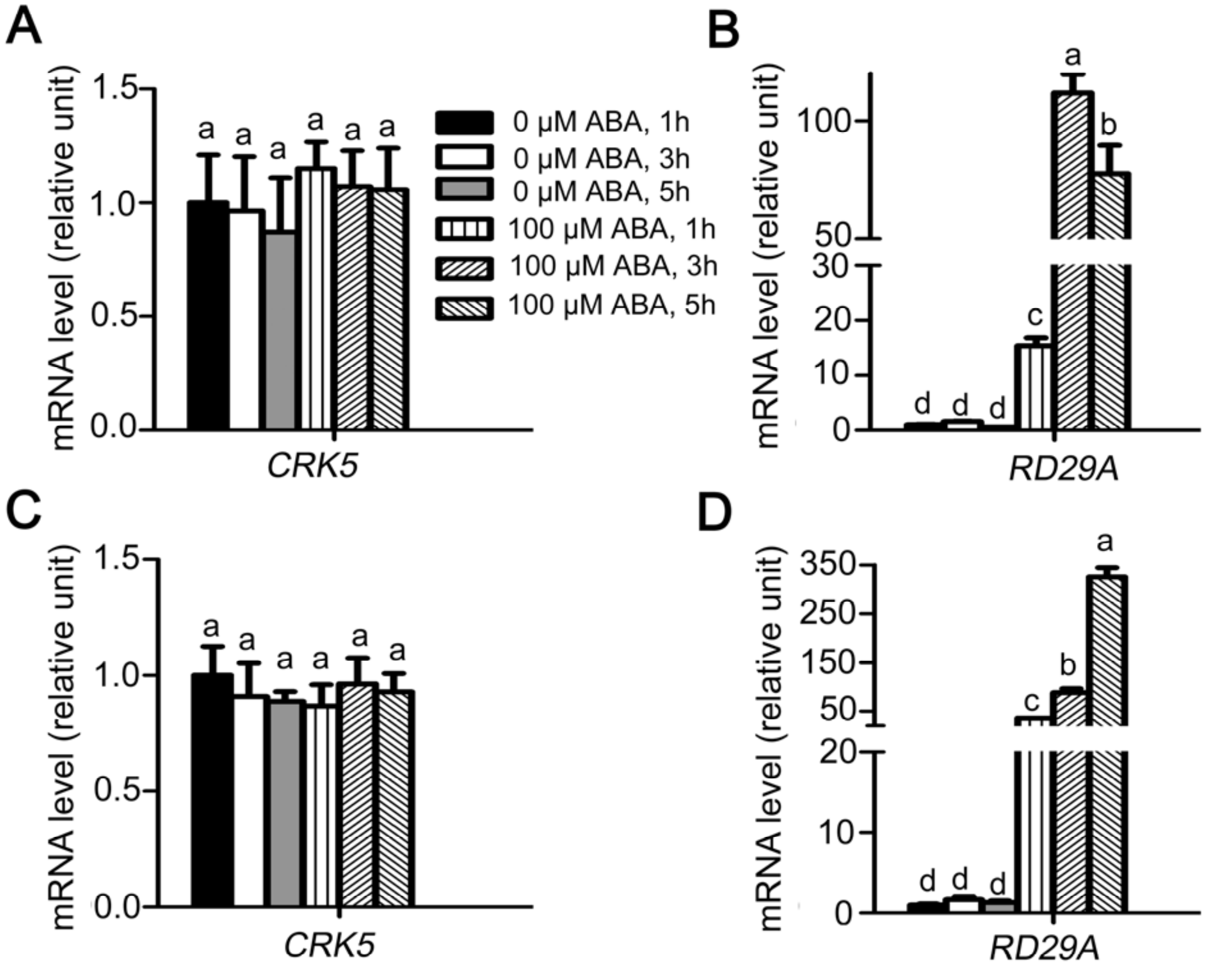

Supplementary Fig. S11. Test of the effects of ABA treatment on *CRK5* gene expression. **(A)-(D)** Two-week-old seedlings grown on MS medium (A and B) or four-week-old plants grown in soil (C and D) were sprayed with the ABA-free (0 μM) or 100 μM (±)ABA-containing solution. The materials were collected at the indicated times and were sampled for RNA extraction. The transcription levels of *CRK5* and *RD29A* were assayed by real-time PCR. Test of the ABA-inducible *RD29* expression was used as a positive control. *Actin2/8* was used as an internal control. Each value is the mean ± SE of three independent experiments, and the letters indicate significant differences at $P < 0.05$ (Duncan's multiple range test).

Supplementary Table S1. PCR primers used in this study.

1. **Primers for identification of the mutants**

Primer Name	Sequence（5'-3'）
**LBa1**	TGGTTCACGTAGTGGGCCATCG
***crk5-1*-LP**	GAAAGTTACGGCGGACCAATC
***crk5-1*-RP**	TGTGAATTAAACAGAGCCATCCC
***crk5-2*-LP**	CGGAAAGGAAGGAGTTGAAAC
***crk5-2*-RP**	TTTTCCGGAAACAGATTCATG
***crk19-1*-LP**	TTGTTGATGCTTTGACCACTG
***crk19-1*-RP**	GCCTTGATCACTTCCTCTTTG
***crk19-2*-LP**	TTGTTGATGCTTTGACCACTG
***crk19-2*-RP**	CTTGTCTACGAGTTTGTGCCC

2. **Primers for generating the transgenic lines and the mutagenesis of *CRK5*^{K372E}**

Primer Name	Sequence（5'-3'）
**CRK5-GFP-F:**	CGGGATCCATGTCTGCTTATACCTCATTAAAC
**CRK5-GFP-R:**	GGGGTACC ACGAGGAGCTAAAATAGTAATCG
**K372E-Middle-F:**	AGTTGCCGTGGAGAGACTATCGAAAACATCAGGA
**K372E-Middle-R:**	TCCTGATGTTTTCGATAGTCTCTCCACGGCAACT
**CRK4-GFP-F:**	TCCCCCGGGATGTCTTTCTTCTGGCTTTTTC
**CRK4-GFP-R:**	GGGGTACCACGAGGAGTTACATTAGTAAT
**CRK19-GFP-F:**	TCCCCCGGGATGTCTTCTCTGATCTCTTTC
**CRK19-GFP-R:**	GGGGTACCACGAGGAGTTACACGAGTAATG
**CRK20-GFP-F:**	TCCCCCGGGATGTCTTCTCTGATCTGTTTC
**CRK20-GFP-R:**	GGGGTACCACGAGGAGCTACACTAGTAATG
**CRK5-GUS-F:**	TCCCCCGGG GGCCAGTAAAGTCTTGCTCCTA
**CRK5-GUS-R:**	ACGCGTCGAC TATCCAATTTCTTCACCTTTCTTC

3. **Primers for yeast one-hybrid assay.**

Primer Name	Sequence（5'-3'）
CRK5-pHIS2-F:	TCCCCCGGG GGCCAGTAAAGTCTTGCTCCTAT
CRK5-pHIS2-R:	CGACGCGT TATCCAATTTCTTCACCTTTCTTCT
WRKY18-AD-F	GGAATTCCATATG ATGGACGGTTCTTCGTTTCTC
WRKY18-AD-R	CCGCTCGAGG TCATGTTCTAGATTGCTCCATT
WRKY40-AD-F	GGAATTCCATATG ATGGATCAGTACTCATCCTCTTT
WRKY40-AD-R	CCGCTCGAGG CTATTTCTCGGTATGATTCTGTT
WRKY60-AD-F	GGAATTCCATATGATGGACTATGATCCCAACACCAA
WRKY60-AD-R	CCGCTCGAGG TCATGTTCTTGAATGCTCTA

4. **Primers for transient transformation of tobacco leaves**

Primer Name	Sequence（5'-3'）
CRK5-LUC-F:	TCCCCCGGG GGCCAGTAAAGTCTTGCTCCTA
CRK5-LUC-R:	ACGCGTCGAC TATCCAATTTCTTCACCTTTCTTC
WRKY18-Flag-F	TCCCCCGGG ATGGACGGTTCTTCGTTTCTCG
WRKY18-Flag-R	GGGGTACC TGTTCTAGATTGCTCCATTAAC
WRKY40-Flag-F	GCTCTAGA ATGGATCAGTACTCATCCTCTTTGGT
WRKY40-Flag-R	GGGGTACC TTTCTCGGTATGATTCTGTTGATACAAT
WRKY60-Flag-F	GCTCTAGA ATGGACTATGATCCCAACACCAA
WRKY60-Flag-R	GGGGTACCTGTTCTTGAATGCTCTATCAATC

5. **Primers for production of recombinant protein**

Primer Name	Sequence（5'-3'）
WRKY18-PET48B-F	GGAATTCT ATGGACGGTTCTTCGTTTCTC
WRKY18-PET48B-R	ACGCGTCGAC TCATGTTCTAGATTGCTCCATT
WRKY40-PET48B-F	GGAATTCT ATGGATCAGTACTCATCCTCTTTGGT
WRKY40-PET48B-R	ACGCGTCGAC CTATTTCTCGGTATGATTCTGTT
WRKY60-PET48B-F	GGAATTCT ATGGACTATGATCCCAACACCAA

WRKY60-PET48B-R	ACGCGTCGAC TCATGTTCTTGAATGCTCTA
CRK5-GST-F	CGGGATCCATTGAAGCTGCAACAGATAAGTT
CRK5-GST-R	CCGCTCGAG TTAACGAGGAGCTAAAATAGTA

6. Primers for identification of the precise insertion point of transgenic lines by tail-PCR

Primer Name	Sequence（5’-3’）
Specific primer1	TACTCGCCGATAGTGGAAACCG
Specific primer2	AAAGAAATAGAGTAGATGCCGACCG
Specific primer3	TACTCGCCGATAGTGGAAACCG
Random primer1	NTCGASTWTSGWGTT
Random primer2	NGTCGASWGANAWGAA
Random primer3	WGTGNAGWANCANAGA
Random primer4	AGWGNAGWANCAWAGG
	W = A or T; S = C or G; N = A, C, G or T
CRK5OE-1-F	ACATGAAACTTCAAACCACCCATTT
CRK5OE-1-R	GGTGATTTCGGTCTATCGTCTTTTTT
CRK5OE-2-F	GAGTTTATGTAATGTTTGGGAGTTG
CRK5OE-2-R	AAGCCATCGTAATAGAGTCAGTTTT
ABI2OE-F	AGTAGTGGTAGCAAGCGAGGCA
ABI2OE-R	CCCGAAAGTGGTGAATGAAGTG

7. Primers for qRT-PCR

Primer Name	Sequence（5’-3’）
qPCR-CRK5-F	TTCAACAAAGTTGGAGGAAGA
qPCR-CRK5-R	ACACAAATAAGAACTGAGATAGCG
qPCR-CRK4-F	TCTACAATGAAACCGCCACT
qPCR-CRK4-R	CCCGGAGACTAAAGAAAGCT
qPCR-CRK19-F	GACTGTCTGAAAATAGGCATC
qPCR-CRK19-R	CCTTCTCATTAAGCGTCC

qPCR-CRK20-F	CTGCCTACAACAAAGCATCA
qPCR-CRK20-R	GGTATCGCTCCAATCGTCT
qPCR-Actin2/8-F	GGTAACATTGTGCTCAGTGGTGG
qPCR-Actin2/8-R	AACGACCTTAATCTTCATGCTGC
qPCR-RD29A -F	ATCACTTGGCTCCACTGTTGTTC
qPCR-RD29A-R	ACAAAACACACATAAACATCCAAAGT
qPCR-RD29B-F	CTTGGCACCACCGTTGGGACTA
qPCR-RD29B -R	TCAGTTCCCA GAATCTTGAACT
qPCR-RAB18-F	CAGCAGCAGTATGACGAGTA
qPCR-RAB18-R	CAGTTCCAAAGCCTTCAGTC
qPCR-DREB2A-F	AAGGTAAAGGAGGACCAGAG
qPCR-DREB2A -R	ACACAACCAGGAGTCTCAAC
qPCR-ABI3-F	ACCCAAATCGGAGAAACCTGTG
qPCR-ABI3-R	GAGGTTCCGATGTCTTCCATGG
qPCR-ABI5-F	CAATAAGAGAGGGATAGCGAACGAG
qPCR-ABI5-R	CGTCCATTGCTGTCTCCTCCA
qPCR-Em1-F	CAAAGCAACTGAGCAGAGAAGAGC
qPCR-Em1-R	CCTCCCTTGCTCCTTCCTTCA
qPCR-Em6-F	CAGCAGATGGGACGCAAAGG
qPCR-Em6-R	TATTACATCCGTGTGGGGAAGTTTG
qPCR-SnRK2.2-F	ATATGCCATCGGGATCTGAA
qPCR-SnRK2.2-R	TTGGTTGGGAATGAAGAACAG
